# Critical heat flux maxima during boiling crisis on textured surfaces

**DOI:** 10.1038/ncomms9247

**Published:** 2015-09-08

**Authors:** Navdeep Singh Dhillon, Jacopo Buongiorno, Kripa K. Varanasi

**Affiliations:** 1 Department of Mechanical Engineering, Massachusetts Institute of Technology, 35-209, 77 Massachusetts Avenue, Cambridge, Massachusetts 02139, USA; 2 Department of Nuclear Science and Engineering, Massachusetts Institute of Technology, 24-206, 77 Massachusetts Avenue, Cambridge, Massachusetts 02139, USA

## Abstract

Enhancing the critical heat flux (CHF) of industrial boilers by surface texturing can lead to substantial energy savings and global reduction in greenhouse gas emissions, but fundamentally this phenomenon is not well understood. Prior studies on boiling crisis indicate that CHF monotonically increases with increasing texture density. Here we report on the existence of maxima in CHF enhancement at intermediate texture density using measurements on parametrically designed plain and nano-textured micropillar surfaces. Using high-speed optical and infrared imaging, we study the dynamics of dry spot heating and rewetting phenomena and reveal that the dry spot heating timescale is of the same order as that of the gravity and liquid imbibition-induced dry spot rewetting timescale. Based on these insights, we develop a coupled thermal-hydraulic model that relates CHF enhancement to rewetting of a hot dry spot on the boiling surface, thereby revealing the mechanism governing the hitherto unknown CHF enhancement maxima.

## Abstract (web summary)

Cooling hot surfaces by boiling water is widely practiced, but the amount of heat transfer is normally constrained by vapour layer formation at sufficiently high temperatures. Here, the authors report the maximum in the critical heat flux on textured hydrophilic surfaces at an intermediate texture density.

## Graphical abstract

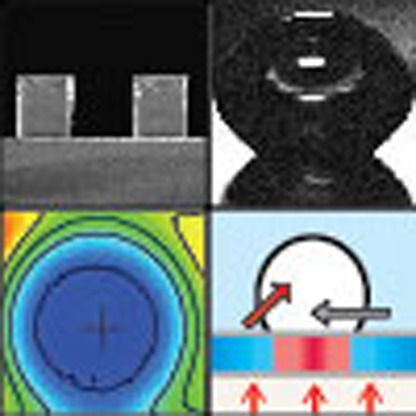

Boiling is a ubiquitous phenomenon of vital importance in a broad range of industries such as power generation, desalination, chemical processing, refrigeration and electronics thermal management^1^,^2^,^3^,^4^,^5^. Nucleate boiling is an efficient way of transferring heat; for a given difference between the heat transfer surface and the fluid temperatures (the so-called wall superheat), nucleate boiling can dissipate up to two orders of magnitude larger heat fluxes compared with liquid convection^6^. The heat transfer coefficient and compactness of boiling heat exchangers increases with increasing heat flux, but beyond a critical value of the heat flux (or CHF) a vapour layer^1^,^5^ develops between the liquid and the solid surface, thus severely limiting heat transfer, a phenomenon referred to as the boiling crisis. The resulting film boiling regime leads to an abrupt large rise in surface temperatures, which can lead to catastrophic burnout of the boiling surface. CHF is the chief thermal performance limiter in light water-cooled nuclear reactors^7^, and the main reason why industrial boilers are forced to operate at significant heat flux safety margins. Enhancing CHF can lead to substantial cost savings in terms of reduced capital investment, and significant reductions in carbon emissions.

Despite being an active area of research for over half a century, the boiling crisis is one of the least understood among known thermal and hydraulic instabilities^8^. Prior studies on increasing CHF include modification of fluid and surface properties^1^,^5^,^9^, and use of active techniques such as applied electric fields^10^,^11^. Surface modifications are particularly attractive because often there are constraints on selection of other fluids and operational conditions. Although most previous studies have identified surface roughness, wettability and porosity as important properties affecting CHF, the physical mechanisms governing the phenomenon remain largely unclear, with widely varying hypothetical explanations such as hydrodynamic instabilities^12^,^13^, liquid macrolayers^14^,^15^, vapour recoil^6^,^16^,^17^ and surface wetting^18^,^19^,^20^. These studies, although informative, have mostly been limited to randomly textured surfaces, such as particle coatings^12^,^14^, nanowires^21^, graphene^22^ and anodized porous materials^18^,^19^, and therefore do not provide a clear understanding of the effect of texture on CHF. The conventional hydrodynamic instability formulation of CHF^23^ does not fundamentally account for surface effects, whereas the mostly empirical macrolayer dryout theory^24^ is in question due to recent experimental studies^25^ challenging the existence of the macrolayer. A static porous media flow approach similar to conventional heat pipe analysis^26^ has been attempted but found unable to explain CHF enhancement on porous surfaces^27^. Other recent studies employing parametric textures^16^,^17^ have combined a static force balance approach at the liquid–vapour contact line of a bubble^28^ with roughness parameters to predict a monotonic increase in CHF with surface texture density. Although this approach to explaining texture-induced CHF enhancement is promising, it does not take into account the dynamics of the contact line, which is essential to capture the physics of the phenomena. After experiments^18^,^19^,^20^ indicated that it is surface wetting and not roughness *per se* that enhances CHF, more recent studies^29^,^30^ (especially Rahman *et al*.^29^) have come up with strong correlations between experimental data on CHF and liquid imbibition (or wicking) into the microstructures. However, they propose no physical mechanism detailing the role of liquid imbibition in the boiling crisis, adding essentially a data-correlated term to existing CHF models^23^,^28^.

In this paper, we present data showing that the CHF of a textured hydrophilic surface neither increases monotonically with texture density^16^,^17^ nor can its trend be captured accurately using an arbitrary liquid imbibition correlation^29^,^30^. As the static force balance approach^16^,^17^ appears inadequate in explaining our data, we study the interaction of texture-induced liquid microflows with the thermal behaviour of the boiling surface. Supported by high-speed optical and infrared imaging analysis of boiling, we propose and justify a dynamic mechanism for surface texture-induced CHF enhancement based on a coupling between capillary surface rewetting and thermal thin-film evaporation.

## Results

### Pool boiling experiments on textured surfaces

**Figure 1:**
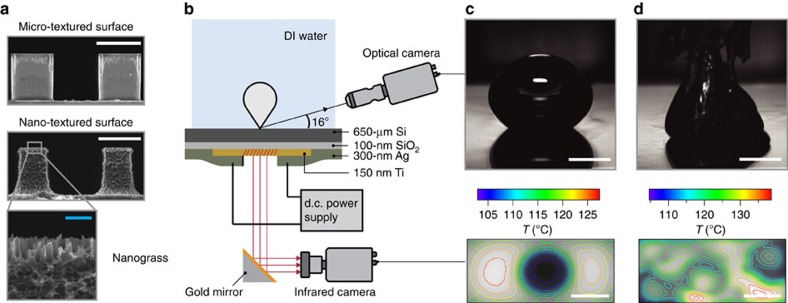
Methodology of boiling experiments. (**a**) Parametrically designed micro- and nano-textured boiling surfaces. The micro-textured surface is composed of plain square micropillars (width *a*=10 μm and height *h*=12.75 μm) etched onto a 650-μm-thick silicon substrate. In the nano-textured surface, the micropillars are covered by nanograss, which is a nano-texture with a length scale of ∼100 nm. Blue scale bar, 1 μm. White scale bar, 10 μm. (**b**) Boiling experimental apparatus with heating element and high-speed optical/infrared data acquisition setup. Current passed through a 1 × 2 cm^2^ Ti thin-film heater on the backside of the silicon substrate induces boiling on its textured top surface. Temperature distribution on the boiling surface is calculated by acquiring an infrared image of the Ti heater from the backside, and correcting for temperature drop across the sample. (**c**) Optical image of a single bubble on the micro-textured surface with 5 μm-spaced micropillars and the corresponding surface temperature at a low heat flux of *q*''=20 W cm^−2^. Scale bar, 5 mm. (**d**) Optical image of boiling and surface temperature distribution at a high heat flux close to CHF (*q*''=165 W cm^−2^, CHF=169 W cm^−2^). Scale bar, 5 mm.

To understand the effect of different surface-texture length scales on the boiling crisis, we studied parametrically designed micro- and nano-textured boiling surfaces. The textures were created by pattern-etching a square array of *a*∼10 μm wide and *h*∼12.75 μm high square micropillars on a 650-μm-thick silicon substrate. Silicon was chosen as the test surface due to its compatibility with standard cleanroom microfabrication processes such as deep reactive ion etching and piranha cleaning. The micro-textured surface is composed of plain micropillars, whereas the nano-textured surface is composed of micropillars covered with an ∼100 nm length scale nano-texture called nanograss (Fig. 1a and Supplementary Figs 1 and 2). Noting that capillary wetting will have an important role in the liquid micro-flows on the boiling surface, the pitch of the textures was systematically altered by varying the spacing *b* between the micropillars. A 1 × 2 cm^2^ titanium thin-film heater was patterned on the electrically insulated backside of the 5 × 5 cm^2^ silicon samples (Fig. 1b and Supplementary Fig. 3) to precisely define a 2-cm^2^ boiling area on their top surface, covered completely by the texture. The size of the heated area was chosen to be large enough to mimic boiling behaviour on an infinite boiling surface^31^,^32^, and it can be shown that conduction heat losses from the boiling area are negligible (Supplementary Note 1). Deionized (DI) water was boiled on the textured surfaces by passing DC electrical current through the titanium thin-film heater inside a pool-boiling chamber maintained at 100 °C and 1 atm. A high-speed optical camera was used to observe boiling on the top surface, and a high-speed infrared camera was used to measure the temperature of the substrate from the backside (Fig. 1b,c). See the Methods section for more details on sample fabrication and the experimental procedure.

### Effect of surface texture on CHF

**Figure 2:**
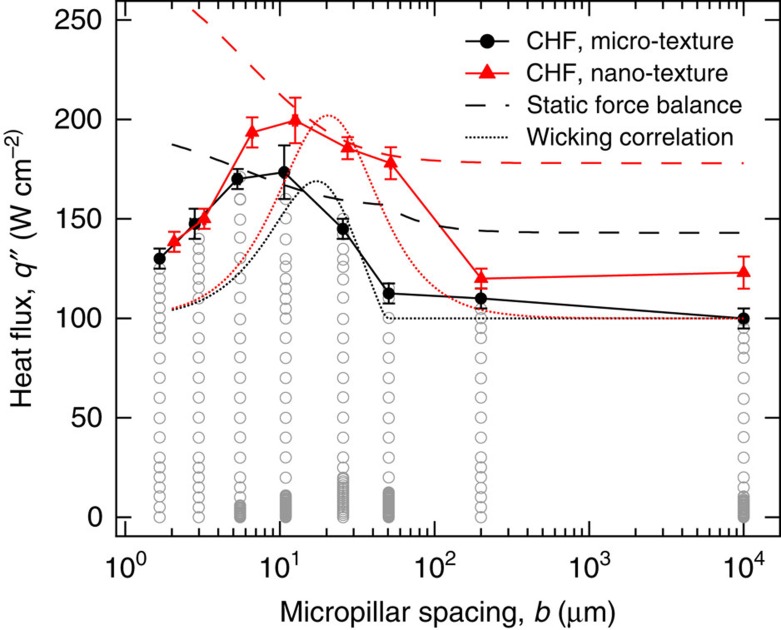
Effect of surface texture on critical heat flux. Plot of CHF versus micropillar spacing *b* for both the micro- and nano-textured surfaces. CHF corresponds to the maximum value of applied heat flux *q*'' (data points shown for the micro-textured surface) that sustained nucleate boiling on the silicon substrate, exceeding which the substrate was damaged by a rapid rise in temperature. The error bar denotes measurement uncertainty and is equal to either the difference of CHF measurements on two nominally similar samples or the maximum heat flux increment (10 W cm^−2^), whichever is greater (Supplementary Fig. 4 and Supplementary Note 2). CHF for both the micro- and nano-textured surfaces initially increases with decreasing micropillar spacing but falls sharply after reaching a maximum value at *b*∼10 μm. The static force balance model^16^,^17^ incorrectly predicts a monotonic increase in CHF with decreasing micropillar spacing, whereas the liquid wetting-based CHF correlation^29^ agrees only qualitatively with the experimental data especially for *b*≤10 μm (Supplementary Note 3).

To characterize the effect of surface texture on the boiling crisis, the heat flux *q*″ applied to the boiling samples was increased from *q*″=0 to *q*″=CHF in maximum 10 W cm^−2^ increments. For each type of surface studied, CHF for two nominally similar samples was measured to obtain an average CHF value and an associated measurement uncertainty, which is equal to either the difference of the two measurements or 10 W cm^−2^, whichever is greater (see Supplementary Fig. 4 and Supplementary Note 2). It was observed that at CHF a localized hot spot develops and subsequently spreads over the entire thin-film heater (Supplementary Movie 1), which either damages the heater or cracks the silicon substrate. The value of CHF for DI water at atmospheric pressure on a flat silicon substrate was measured to be 100±5 W cm^−2^ (Fig. 2 and Table 1), which was taken as the baseline CHF value for this study. A plain silicon wafer with just the nanograss has only a slightly higher CHF at 123±8 W cm^−2^. For widely spaced micropillars (*b*=200 μm), the CHF of both the micro- and nano-textured surfaces is almost equal to the corresponding surface without the micropillars. A further reduction in micropillar spacing leads to a gradual increase in CHF, starting somewhere between *b*=25 μm and *b*=50 μm for the micro-textured surface and between *b*=50 μm and *b*=200 μm for the nano-textured surface. This initial result agrees qualitatively with the static force balance model^16^,^17^, which predicts CHF to increase monotonically with an areal surface roughness parameter *r*, defined as the ratio of the actual surface area to the projected surface area. The logic behind their argument is that surface roughness increases the effective length of the three-phase contact line, thereby increasing the capillary forces that help to prevent the boiling crisis by keeping the surface wetted with liquid. For a given measured nanograss roughness of *r*_ng_=3.43 (Supplementary Fig. 2), the roughness of both our micro-textured (*r*_m_=1+4*ah*/(*a*+*b*)^2^) and nano-textured (*r*_n_=*r*_ng_[1+4*ah*/(*a*+*b*)^2^]) surfaces increases monotonically with decreasing micropillar spacing *b*, and therefore one might expect CHF to exhibit a similar monotonic trend.

However, counterintuitively our data reveal that although the CHF initially increases with decreasing micropillar spacing *b*, it then decreases sharply after reaching a maximum value at *b*=10 μm for both the micro-textured (173.5±13.5 W cm^−2^) and nano-textured (199.5±11.5 W cm^−2^) surfaces (Fig. 2). This finding suggests that the effect of surface texture on CHF cannot be modelled solely using a static force scaling argument. Other more recent studies^29^,^30^ have attempted to correlate CHF enhancement data to measured liquid imbibition into the surface texture without attempting to justify the underlying physical mechanisms of its relation to the boiling crisis. In Supplementary Note 3, we show that the theoretical expression for CHF based on this purely experimental correlation^29^ does not have a closed form solution. However, for the sake of comparison, we plot a scaled version of this solution in Fig. 2 (by matching the maximum value of CHF) and observe that it agrees qualitatively but not quantitatively with our experimental data, especially at low micropillar spacings. Instead, consideration of the dynamic nature of surface rewetting and its interaction with the concurrent thermal effects is required, as will be done next.

### Boiling crisis hypothesis

**Figure 3:**
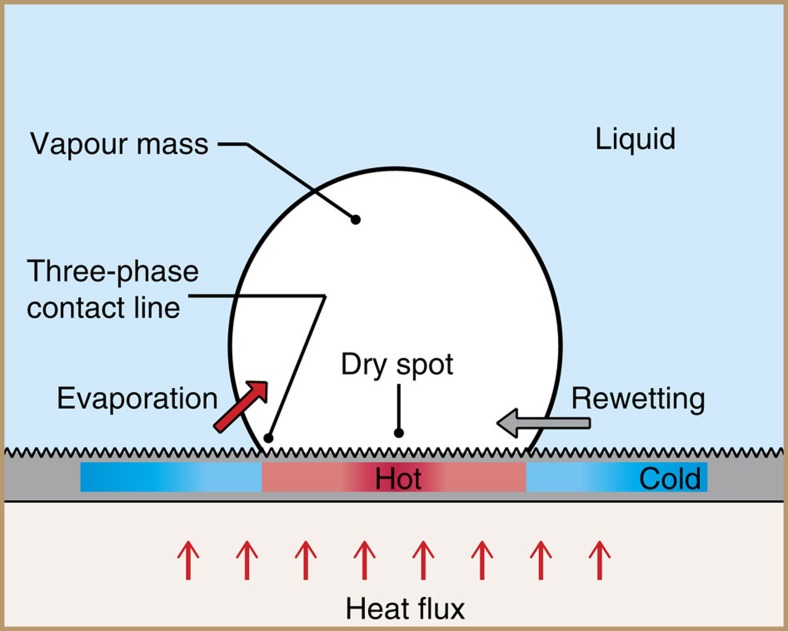
A coupled thermal-hydraulic approach for modelling the boiling crisis. The schematic shows a dry spot formed on the boiling surface underneath a mass of vapour at heat fluxes close to CHF. Substrate temperature at the centre of the dry spot should be higher because of lack of an effective heat removal mechanism. The boiling crisis phenomenon can be understood in terms of competition between liquid rewetting of the dry spot because of gravitational and capillary forces and enhanced liquid evaporation because of elevated surface temperatures in the interior of the dry spot.

It is known that at heat fluxes close to CHF dry spots are continuously forming on the boiling surface and getting rewetted by the surrounding liquid^6^,^8^,^25^,^33^,^34^,^35^. As also indicated by our infrared visualization of the boiling substrate, the boiling crisis is characterized by one or more of these localized dry spots spreading irreversibly and enveloping the entire heated surface in a continuous vapour layer. In the process, the substrate temperature escalates rapidly as the mechanism of heat transfer from the substrate to the liquid transitions from liquid convection/evaporation to conduction/radiative transport through the vapour layer. Challenging the assumptions of the conventional hydrodynamic theory^23^, which completely ignore the boiling substrate, researchers have long suspected that these dry spots have an important role in triggering the boiling crisis^36^. However, an accurate mechanistic model for CHF based on this concept is not available, primarily due to difficulties associated with experimental visualization of the phenomena. Taking advantage of the parametric dependence of CHF on surface texture and aided by infrared thermal visualization of the substrate, we develop a coherent physical model for CHF by analysing a random dry spot formed on the boiling surface underneath a mass of vapour at heat fluxes close to CHF (Fig. 3). Most of the evaporation into the vapour mass occurs from the liquid–vapour interface near the three-phase contact line, and is roughly proportional to the surface superheat temperature^37^,^38^. This liquid evaporation results in the motion of the liquid–vapour interface away from the dry spot because of mass and momentum conservation. Under normal circumstances, the surrounding liquid moves in to rewet the surface under the influence of gravitational and capillary forces, and the dry spot disappears. However, at high heat fluxes the temperature in the interior of the dry spot may rise rapidly due to the incoming heat flux and the absence of evaporative/liquid convective cooling. Therefore, we hypothesize that the boiling crisis is triggered by the inability of the surrounding liquid to rewet a localized dry spot on the boiling surface because of a competition with high rates of evaporation precipitated by elevated temperatures in the interior of the dry spot. We propose that the effect of surface texture on CHF can be understood by looking at the role it has in the rewetting of a dry spot.

### Dry spot thermal characteristics

**Figure 4:**
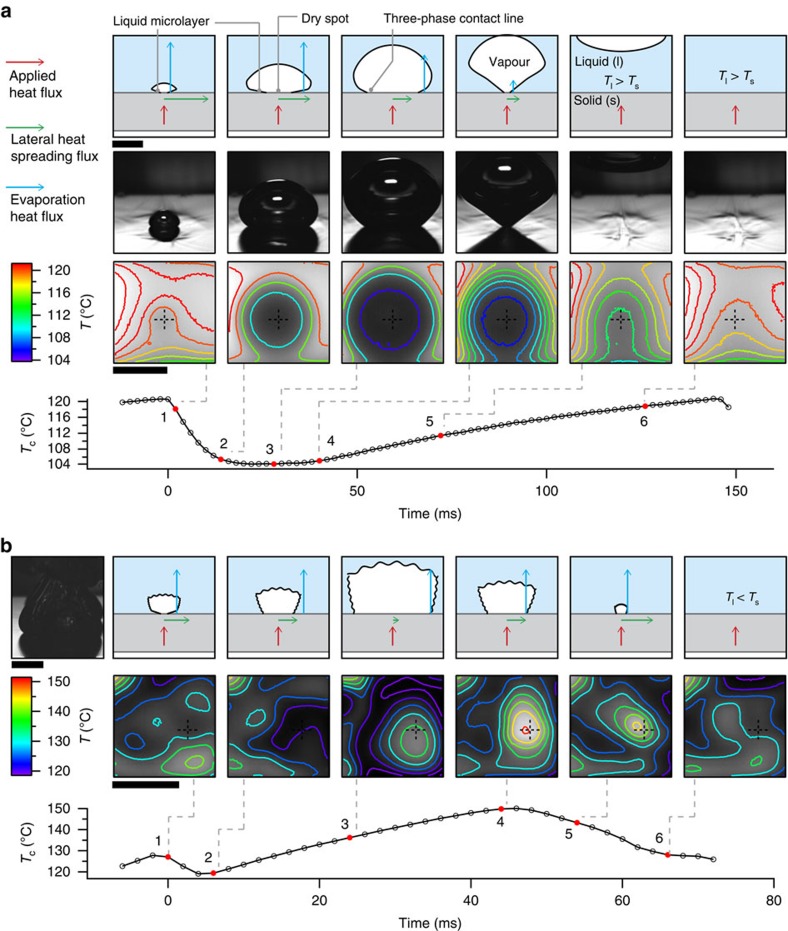
Formation of a hot dry spot at CHF. Schematic representation and optical/infrared visualization of boiling at low and high heat fluxes on the micro-textured surface with 10-μm-spaced micropillars. (**a**) At a low applied heat flux of 20 W cm^−2^, a single periodic bubble forms and departs from the surface. The dry spot formed under the bubble is colder than the surrounding fluid, cooled initially by evaporation of the liquid microlayer^37^,^38^ and later by lateral diffusion of heat to the evaporating three-phase contact line. Plot shows variation with time of substrate temperature at the centre of the bubble *T*_c_ (cross mark on temperature map). Scale bar, 5 mm. (**b**) At a high heat flux of 180 W cm^−2^ close to CHF (185 W cm^−2^), the formation and collapse of a ‘hot' dry spot is observed on the boiling surface. The dry spot, identified by a hot interior and an evaporation-induced cold periphery, grows to a maximum radius of ∼2–3 mm before being rewetted and cooled by the surrounding liquid. The continuous heating of the dry spot during both its growth and collapse can be attributed to the much larger applied heat flux and comparatively low lateral heat diffusion. Scale bar, 5 mm.

To justify our hypothesis for the boiling crisis, we first analysed the thermal behaviour of the boiling substrate at different heat fluxes to identify and characterize a hot dry spot. A real-time temperature map of the boiling substrate and boiling images was obtained using high-speed infrared and optical cameras, respectively, for boiling on the micro-textured surface with 10 μm-spaced micropillars (Fig. 4). It should be mentioned that as the infrared camera can directly measure only the temperature *T*_h_(*x*, *y*) of the infrared-opaque Ti heater on the back of the substrate, we calculate the boiling surface temperature *T*_b_(*x*, *y*) using Fourier's law: *T*_b_(*x*, *y*)=*T*_h_(*x*, *y*)—*q*″·*t*_s_/*k*_s_. Here *q*″ is the applied heat flux, *t*_s_∼0.6 mm the substrate thickness and *k*_s_∼105 W m^−1^ K^−1^ the thermal conductivity of the substrate. There are two approximations associated with this approach, which have a minimal affect on our analysis: (i) Steep spatial temperature variations on the boiling surface (for example, at the three-phase contact line) can get smoothed out over a distance on the order of substrate thickness *t*_s_ (∼0.6 mm), which fortunately is much smaller than the bubble/dry spot diameter we observe (∼5 mm). (ii) Sudden temperature fluctuations on the boiling surface require a thermal diffusion time 
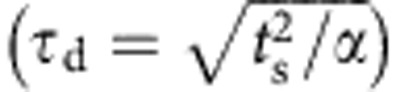
 of ∼3.4 ms to accurately reflect in *T*_b_(*x*, *y*), which again is much smaller than the typical lifetime of a bubble/dry spot (∼50 ms).

At a low heat flux of 20 W cm^−2^, we obtain a single periodic bubble that grows and departs from the surface (Fig. 4a). The bubble does not nucleate until the substrate temperature reaches a critical value (∼120 °C) required for the pressure of vapour trapped in a nucleation cavity to overcome surface tension^39^,^40^. Once the bubble nucleates and starts to grow, there is a rapid drop in substrate temperature at the centre of the bubble *T*_*c*_ because of evaporation of the liquid microlayer^37^,^38^ underneath the bubble (point 1→2). This evaporative cooling effect at the bubble centre is felt to a lesser degree as the three-phase contact line moves farther out because of bubble growth and evaporation of the liquid microlayer formed in the initial stages of bubble growth (inertial growth regime^5^). Note that lateral diffusion of heat away from the centre of the dry spot is inversely proportional to its distance from the evaporating three-phase contact line. The evaporative cooling effect is now countered by substrate heating because of the applied heat flux (points 2→4). After the bubble has departed, the substrate temperature continues to rise steadily due to this incoming heat flux until the nucleation temperature is reached again, and the cycle starts over.

The thermal behaviour of the substrate changes as the applied heat flux is increased. At a heat flux close to CHF (Fig. 4b), the drop in substrate temperature at the centre of a dry spot because of evaporation of the liquid microlayer (points 1→2) is short-lived. After a quick initial drop from the dry spot nucleation temperature (∼128 °C) to a minimum temperature of 120 °C, *T*_*c*_ increases to ∼137 °C as the dry spot grows to its maximum size (points 2→3). This behaviour is quite different from that of the single bubble at low heat fluxes, where there was a substantial drop but almost no rise in *T*_c_ (points 1→3 in Fig. 4a). There are two main reasons for this difference: (i) Average temperature of the surrounding liquid is larger at the high heat flux (∼120 °C) than at the low heat flux (∼104 °C), which keeps the substrate temperature higher, as heat is conducted from the substrate to the liquid. (ii) At higher heat fluxes, the initial liquid microlayer on the surface possibly dries up much faster because of the larger incoming heat flux, thereby preventing further evaporation and drop in substrate temperature. Further, because the incoming heat flux is now much larger than the lateral diffusion of heat, the temperature of the dry spot increases during the rest of its growth phase (points 2→3).

Even as the size of the hot dry spot begins to decrease because of its rewetting by the surrounding liquid, the temperature at its centre increases from *T*_c_∼137 °C to *T*_*c*_∼149 °C (points 3→4 in Fig. 4b), a rise of ∼12 °C. After the dry spot is completely rewetted, its temperature begins to drop because of convective cooling by the lower temperature (∼120 °C) surrounding liquid. Close to CHF, these hot dry spots are constantly forming under bubbles, and have to be rewetted by the surrounding liquid to keep their temperature below a critical value. We hypothesize that above this critical dry spot temperature *T*_crit_, the surrounding liquid is unable to rewet a dry spot, thus precipitating the boiling crisis.

### Liquid imbibition into surface textures

**Figure 5:**
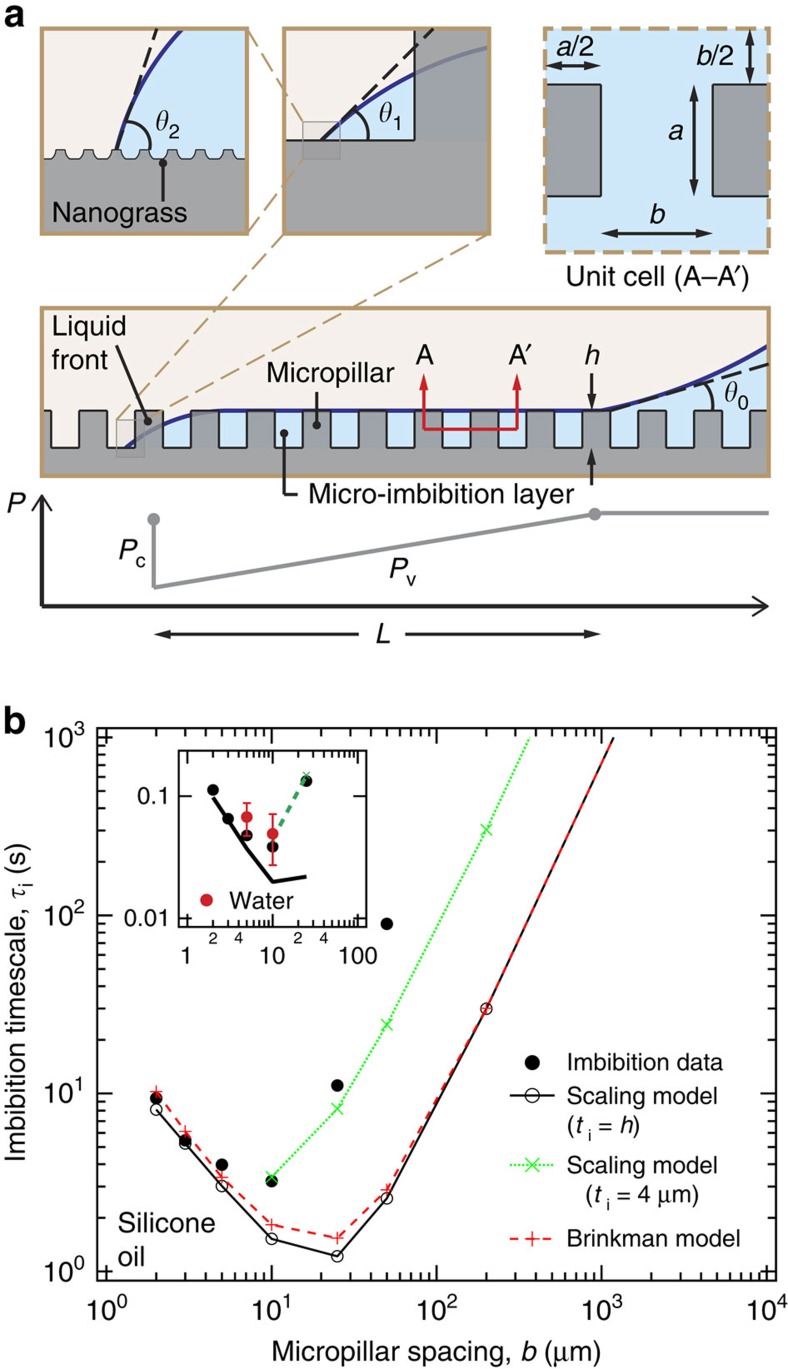
Liquid imbibition into surface micro-textures. (**a**) Owing to capillary suction liquid imbibes into the space between the micropillars forming a micro-imbibition layer. The three-phase contact line of the advancing liquid front exhibits different apparent liquid–solid contact angles at different levels of the surface texture hierarchy: surface level (*θ*_0_), micro-texture level (*θ*_1_), nano-texture level (*θ*_2_). The rate of imbibition is determined by a balance between capillary (*P*_c_) and viscous (*P*_v_) pressures. (**b**) Plot of the experimentally measured and theoretically calculated imbibition time *τ*_i_ for 10 cSt silicone oil to imbibe to a length of 2.5 mm versus the micropillar spacing *b* of the micro-textured surfaces. The predictions of the simpler scaling imbibition model are almost the same as that of a more complex Brinkman model^45^,^49^,^50^. The imbibition models agree with the experimental data corresponding to an imbibed liquid microlayer thickness *t*_i_ equal to the micropillar height *h* for *b*≤10 μm and *t*_i_∼4 μm for *b*>10 μm. The inset shows the oil imbibition results extrapolated to water (Supplementary Note 5) and verified by independent water imbibition experiments (red dots).

Pursuant to our hot dry spot rewetting hypothesis for the boiling crisis and noting that heating of the dry spot should depend primarily on bulk substrate properties, it is clear that the primary role of the surface texture in CHF enhancement is its effect on the rewetting of the dry spot. As we will see later, texture can affect the rewetting of the dry spot by either influencing the bulk movement of the surrounding liquid (sloshing liquid) and/or by imbibing it onto the surface of the dry spot. Here, we focus on the process of liquid imbibition into surface textures, which is dictated by a balance between capillary and viscous forces^41^,^42^,^43^,^44^,^45^,^46^,^47^. The rate of imbibition into the micropillar array of the textured boiling surface is governed by a balance between the capillary pressure (*P*_c_) that develops across the advancing liquid–vapour front of the micro-imbibition layer and the viscous pressure drop (*P*_v_) associated with the flow of liquid between the micropillars (Fig. 5a). Theoretically, the liquid can also imbibe independently into the nanograss ahead of the micro-imbibition liquid front. However, the existence of such a nano-imbibition layer is not feasible because of a much smaller rate of imbibition compared with the trailing micro-imbibition layer, as will be shown below. The role of the nanograss will therefore be limited to modifying the dynamics of the sloshing liquid and the micro-imbibition layer.

To calculate the capillary pressure *P*_c_, we note that the liquid–solid contact angle at different levels of the surface texture hierarchy will be different (Fig. 5a). The nano-texture-level (level 2) liquid–solid contact angle will be equal to the inherent contact angle of liquid on flat solid: *θ*_2_=*θ*. At the micro-texture level (level 1) the apparent liquid–solid contact angle is *θ*_1_=cos^−1^[min(1, *r*_ng_ cos*θ*)], whereas at the topmost surface level it is given by *θ*_0_=cos^−1^[min(1, *r* cos*θ*)], where *r*_ng_ and *r*=*r*_ng_[1+4*ah*/(*a*+*b*)^2^] are the nanograss and overall-texture areal surface roughness, respectively. Considering the work done by the capillary forces on the advancing liquid front, an effective capillary pressure can be calculated using^44^,^45^ *P*_c_=−Δ*E/*Δ*V*, where Δ*E* is the net change in surface energies associated with the imbibition of liquid into a unit cell (Fig. 5a) of the micropillar array of volume Δ*V*=*h*[(*a*+*b*)^2^–*a*^2^] (for details, see Supplementary Note 4). Neglecting wetting of the micropillar tops, the surface energy change is given by Δ*E*=[*b*(2*a*+*b*)+4*ah*]*r*_ng_(*σ*_sl_–*σ*_sv_)+*b*(2*a*+*b*)*σ*_lv_, where *σ*_sl_ is the solid–liquid, *σ*_sv_ the solid–vapour and *σ*_lv_ the liquid–vapour interfacial surface tension. Using the Laplace equation^48^ *σ*_sl_–*σ*_sv_=−*σ*_lv_ cos*θ*, and noting that the capillary force responsible for imbibing the liquid between the micropillars maximizes for *θ*_1_=0, we get


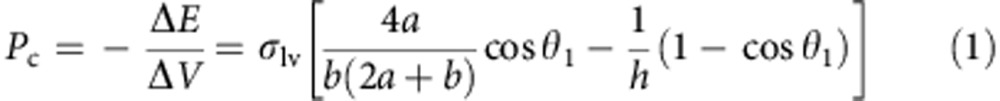


Note that the liquid will imbibe into the micro-textures as long as *P*_c_>0.

The viscous pressure drop associated with the flow of liquid between the micropillars can be written as


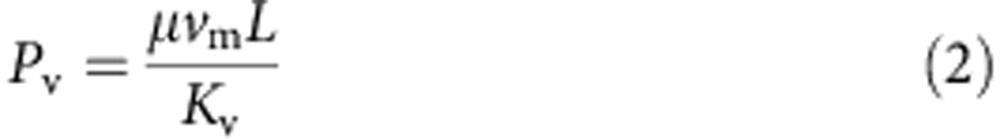


where *μ* is the liquid dynamic viscosity, *v*_m_ is the mean imbibition velocity, *L* is the length of the imbibed layer and *K*_v_ is the in-plane permeability of the surface texture. Using a simple scaling analysis (Supplementary Note 4), we can calculate the surface texture permeability by treating the three-dimensional flow between the micropillars as a combination of flow between parallel plates and free surface flow on a flat plate to get *K*_v_=[3/*h*^2^+24*a*/*b*^2^(*a*+*b*)]^−1^. Equating the capillary pressure and the viscous pressure drop (and ignoring the hydrostatic pressure because the Bond number is low), we can solve for the imbibition time *τ*_i_ required for the liquid to imbibe to a length *L*:


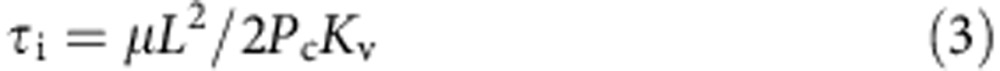


This imbibition model was verified using imbibition experiments, where silicon micro-textured samples were dipped in 10 cSt silicone oil *(θ*∼0) and the imbibing liquid front was tracked using a high-speed camera (Supplementary Movie 2). For experimental accuracy, silicone oil was used instead of water due to its substantially lower vapour pressure at room temperature. Under the intense lighting required for high-speed optical imaging, a substantial amount of evaporation can occur from the thin water film imbibing into the micro-textures. As, for a given texture morphology, the rate of liquid imbibition depends only on the liquid surface tension (*σ*_lv_), liquid viscosity (*μ*) and the liquid–solid contact angle (*θ*), we can easily extrapolate the silicone oil imbibition results to other liquids including water (Supplementary Note 5). Figure 5b plots the measured and theoretically calculated imbibition times for the micro-textured surfaces at different micropillar spacings *b* corresponding to an imbibed length of *L*=2.5 mm. First, we note that the predictions of the scaling imbibition model, which are almost the same as that of a more complex solution^45^,^49^,^50^ based on the Brinkman equation, agree reasonably well with the experimental data at low to moderate micropillar spacings (*b*≤10 μm). For larger micropillar spacings, the thickness of the imbibed liquid microlayer *t*_i_ is expected to be less than the height of the micropillars. Indeed, the model agrees with the data for *b*>10 μm assuming *t*_i_∼4 μm. Second, the occurrence of the minimum in imbibition time *τ*_i_ at the same micropillar spacing (*b*∼10 μm) as the observed CHF maxima indicates that a higher CHF for textured surfaces is in-part due to enhanced rewetting of a hot dry spot by the micro-imbibition layer, which further strengthens our fundamental hypothesis for the boiling crisis. Representing the nanograss using square nanopillars where *a*=*b*=*h*∼100 nm, we can also verify that a liquid will imbibe into the nanograss much slower (*τ*_i,oil_∼180 s) than into the micropillars (*τ*_i,oil_∼1 s). The inset in Fig. 5b shows that the oil imbibition results extrapolated to water (Supplementary Note 5) agree quite well with the imbibition times obtained using independent experiments with water, thereby validating the general applicability of the scaling imbibition model.

### CHF model

**Figure 6:**
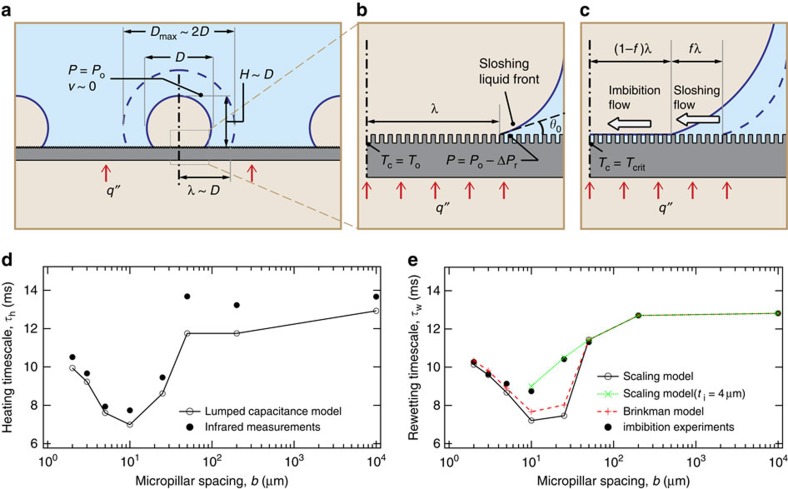
Characteristic timescales for the CHF scaling model. (**a**) An idealized spherical vapour mass formed on the boiling surface at CHF. Corresponding to an average vapour mass diameter *D*, a dry spot of maximum radius *λ*∼*D* can form on the boiling surface. (**b**) At maximum dry spot radius, the initial substrate temperature at the centre of the dry spot is *T*_c_*=T*_o._ Assuming uniform bubble curvature for *θ*_0_=0, the pressure at the sloshing liquid front is equal to the pressure of the comparatively static liquid near the top of the bubble *P*_o_ minus a surface wetting pressure reduction term (Δ*P*_r_) for partially wetting surfaces (*θ*_0_>0). (**c**) The dry spot is rewetted under the combined effect of gravity-induced inward sloshing motion of the surrounding liquid and imbibition of liquid into the surface micro-textures, where *f* is the fractional length of the dry spot rewetted as a result of the sloshing motion. At CHF, the substrate temperature *T*_c_ exceeds the critical dry spot temperature *T*_crit_ before the rewetting liquid front reaches the centre of the dry spot. The heating timescale *τ*_h_ corresponds to the increase in *T*_c_ from *T*_o_ to *T*_crit_, whereas the rewetting timescale *τ*_w_ denotes the time for complete rewetting of the dry spot. (**d**) Plot of the dry spot heating timescale (*τ*_h_) versus micropillar spacing *b* for the micro-textured surfaces at 
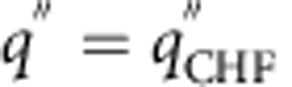
. The calculated timescale is based on a thermal lumped capacitance model, whereas the experimental timescale is based on the maximum substrate temperature ramp rate obtained from infrared measurements. The value of the critical dry spot superheat is assumed to be *T*_crit_—*T*_o_∼12 °C. (**e**) Plot of the dry spot rewetting timescale (*τ*_w_) versus micropillar spacing *b* for the micro-textured surfaces. The calculated timescale is based on the imbibition scaling model, whereas the measured timescale is based on the imbibition experiments (Supplementary Note 5). The contact angle of water on flat silicon was measured to be *θ*∼30° (see Methods).

Having developed an understanding of the surface heating characteristics and the imbibition process in the previous two sections, we now present the dynamic model that predicts the boiling crisis. We focus on an idealized spherical vapour mass with an average diameter *D*∼*D*_max_/2 and average height *H*∼*D*, which results in the formation of a dry spot of maximum radius *λ*∼*D* on the boiling surface (Fig. 6a). At heat fluxes close to CHF, the spacing between neighbouring vapour masses is determined by the Rayleigh–Taylor unstable wavelength^23^,^28^ 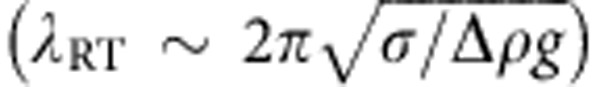. Assuming the maximum vapour mass diameter scales as *D*_max_∼*λ*_RT_/3, its average diameter and the maximum dry spot radius should be proportional to the Laplace length 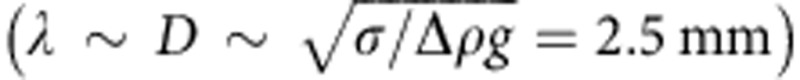. This assumption is supported by our infrared visualization study (Fig. 4b), where a characteristic dry spot is seen to grow to a maximum radius of ∼2–3 mm. Further, we assume that the initial substrate temperature at the centre of the dry spot (*T*_c_) is equal to *T*_o_. As our hypothesis relates the boiling crisis to rewetting of a hot dry spot on the boiling surface, we are interested in the following two timescales: (i) a heating timescale *τ*_h_, in which the temperature at the centre of a fully grown dry spot increases from *T*_o_ to the critical dry spot temperature *T*_crit_, and (ii) a rewetting timescale *τ*_w_, in which the surrounding liquid is able to completely rewet the dry spot. Below, we will postulate that the boiling crisis occurs when *τ*_h_<*τ*_w_.

For a thin boiling substrate, the heating timescale *τ*_h_ can be estimated using a one-dimensional thermal lumped capacitance model. Ignoring any lateral conduction of heat in the thin solid substrate 
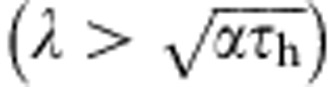
 and assuming no significant temperature gradient along its thickness 
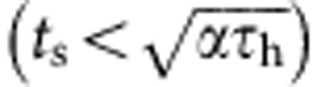
, the heating timescale can be calculated using energy conservation:


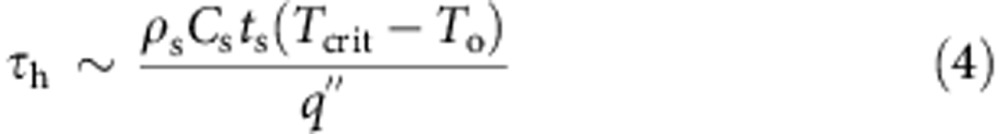


Here 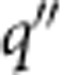 is the applied heat flux, *ρ*_s_ is the density, *C*_s_ the specific heat and *t*_s_ the thickness of the substrate. To determine *τ*_h_, we need a value for *T*_crit_–*T*_o_, the permissible rise in substrate temperature at the centre of the dry spot during its rewetting phase, which we call the critical dry spot superheat. Previously, we saw that at a heat flux close to CHF, the dry spot centre temperature of the micro-textured surface with 10-μm-spaced micropillars, rose from ∼137 °C to a maximum value of ∼149 °C (points 3→4 in Fig. 4b). Similar analysis conducted for dry spots on other samples showed a similar behaviour and values of *T*_o_ and *T*_crit_ of the same order. Based on this observation let us assume that *T*_crit_–*T*_o_∼12 °C for all the boiling samples, noting that the presence of surface textures should have no influence on the sensible heating of the substrate. The plot in Fig. 6d shows the variation of the calculated lumped capacitance-based heating timescale with micropillar spacing *b* for the micro-textured surfaces at 
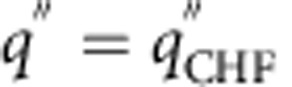
. Also plotted are the minimum experimentally determined heating timescales for these samples based on the infrared surface temperature ramp rate measurements. We observe that the lumped capacitance analysis is able to correctly capture the physics of substrate heating. The average heating timescale is on the order of 10 ms, and as expected from the corresponding CHF data, it exhibits a minimum value at an intermediate micropillar spacing of *b*=10 μm. Using *τ*_h_∼10 ms, we can confirm that our initial modelling assumptions were reasonable in that 0.6 mm<0.8 mm<2.5 mm 
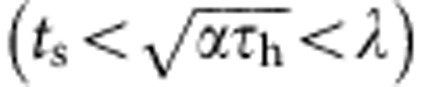
.

At the same time that the substrate temperature is increasing, the surrounding liquid moves in to rewet the dry spot under the influence of gravity and aided by the micro-imbibition layer that develops between the micropillars (Fig. 6b,c). The boiling crisis is avoided if the surrounding liquid reaches the centre of the dry spot before its temperature exceeds the critical dry spot superheat. For calculating the rewetting timescale *τ*_w_, we employ quasi-steady-state scaling analysis to simultaneously account for the roles played by gravity-induced inward sloshing motion of the surrounding liquid and imbibition of liquid into the surface micro-textures. Although, as will be shown below, the sloshing liquid motion indirectly affects the dynamics of liquid imbibition, it is important to note that these are two completely distinct flow mechanisms. Whereas imbibition between the micropillars is treated as viscous flow, the inward sloshing motion of the bulk liquid can be approximated as inviscid, incompressible and irrotational (Bernoulli's flow^51^). Let us assume that during the time the rewetting liquid front travels a distance *λ* to reach the centre of the dry spot, the sloshing liquid front has travelled a distance *fλ* (Fig. 6c). Assuming a uniform radius of curvature for the vapour mass on perfectly wetting surfaces (*θ*_*o*_=0) and uniform vapour pressure inside it, the pressure at the base of the sloshing liquid front should be equal to the pressure at the top of the bubble minus a surface wetting-related pressure reduction term Δ*P*_r_ for partially wetting surfaces (*θ*_0_>0). Starting from the comparatively stationary liquid near the top of the bubble, the average velocity of the sloshing liquid front *v*_*g*_ can be obtained using the Bernoulli's equation^51^: 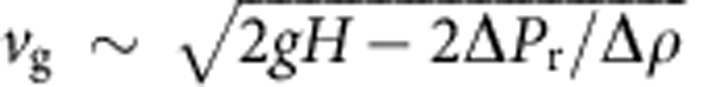. The surface wetting pressure reduction term should scale with the net increase in surface energy per unit area associated with the advancing sloshing front and inversely with its height: Δ*P*_r_∼2*σ*(1–cos*θ*_0_)/*H*. Noting that the effect of Δ*P*_r_ is limited to the scenario where Δ*P*_r_<<Δ*ρgH* (for hydrophobic surfaces with large Δ*P*_r_ the sloshing liquid can just roll onto the surface), we can calculate the dry spot rewetting timescale as follows:


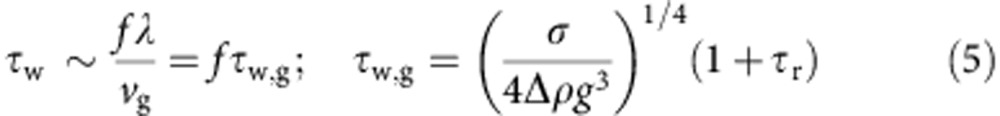


where *τ*_w,g_ denotes the gravity-induced component of the rewetting timescale and the non-dimensional term *τ*_r_=Δ*P*_r_/2Δ*ρgD* accounts for the resistance of partially wetting surfaces to rewetting by the sloshing liquid front. In the absence of liquid imbibition, the dry spot is wetted purely by gravity, in which case *f*=1 and *τ*_w_=*τ*_w,g_. Substituting water properties at 100 °C, we get a value of the gravity induced rewetting timescale for flat silicon of the same order (∼13 ms) as the corresponding heating timescale (Fig. 6d).

If the surface texture is favourable to the formation of a micro-imbibition layer (*P*_c_>0) and the average imbibition liquid front velocity *v*_i_ is larger than *v*_g_, then part of the dry spot is rewetted by this imbibed layer (Fig. 6c). In this scenario, the average velocity of the imbibition liquid front can be calculated using the capillary and viscous pressures given by equations (1) and (2) while noting that the viscous pressure drop in this case only acts along a length (1–*f*)*λ*:


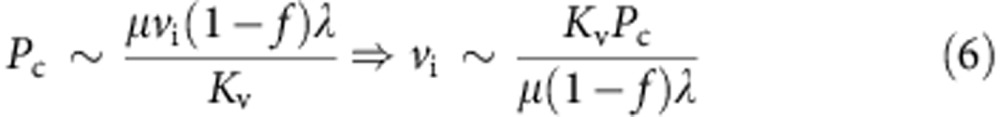


As the imbibition liquid front travels ahead of the sloshing liquid front, it travels the entire length of the dry spot and so the rewetting timescale can be expressed as:


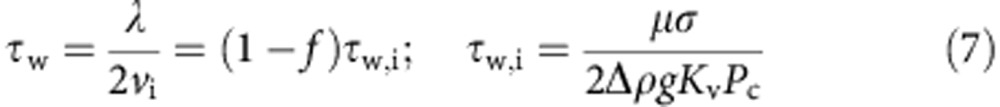


where *τ*_w,i_ denotes the imbibition-induced component of the rewetting timescale. By comparing the rewetting timescales obtained from equations (5) and (7), we find that *f*=*τ*_w,i_/(*τ*_w,g_+*τ*_w,i_). The rewetting timescale can therefore be written as:


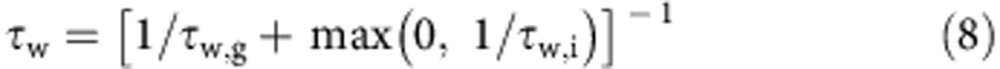


where the max operator is used to preclude a negative contribution to the rewetting timescale in cases where the capillary pressure *P*_c_ is negative (non-imbibing textures). In Fig. 6e, we plot this calculated rewetting timescale versus micropillar spacing *b* for the micro-textured surfaces and see that it agrees quite well with an experimental timescale obtained by extrapolating the silicone oil imbibition results to water (Supplementary Note 5) corresponding to a measured silicon–water equilibrium contact angle of *θ*∼30° (see Methods and Supplementary Fig. 5). It is interesting to note that the dry spot rewetting timescale *τ*_w_ exhibits exactly the same trend as the heating timescale *τ*_h_, strengthening our hypothesis that the phenomena of boiling crisis is dictated by a competition between the heating and rewetting of a dry spot on the boiling surface.

Now that we have obtained expressions for the dry spot heating and rewetting timescales, and verified them against experimental data, the value of CHF can be obtained by equating them using equations (4) and (8):


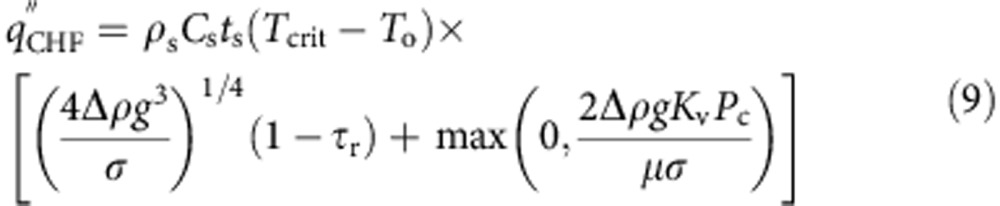


**Figure 7:**
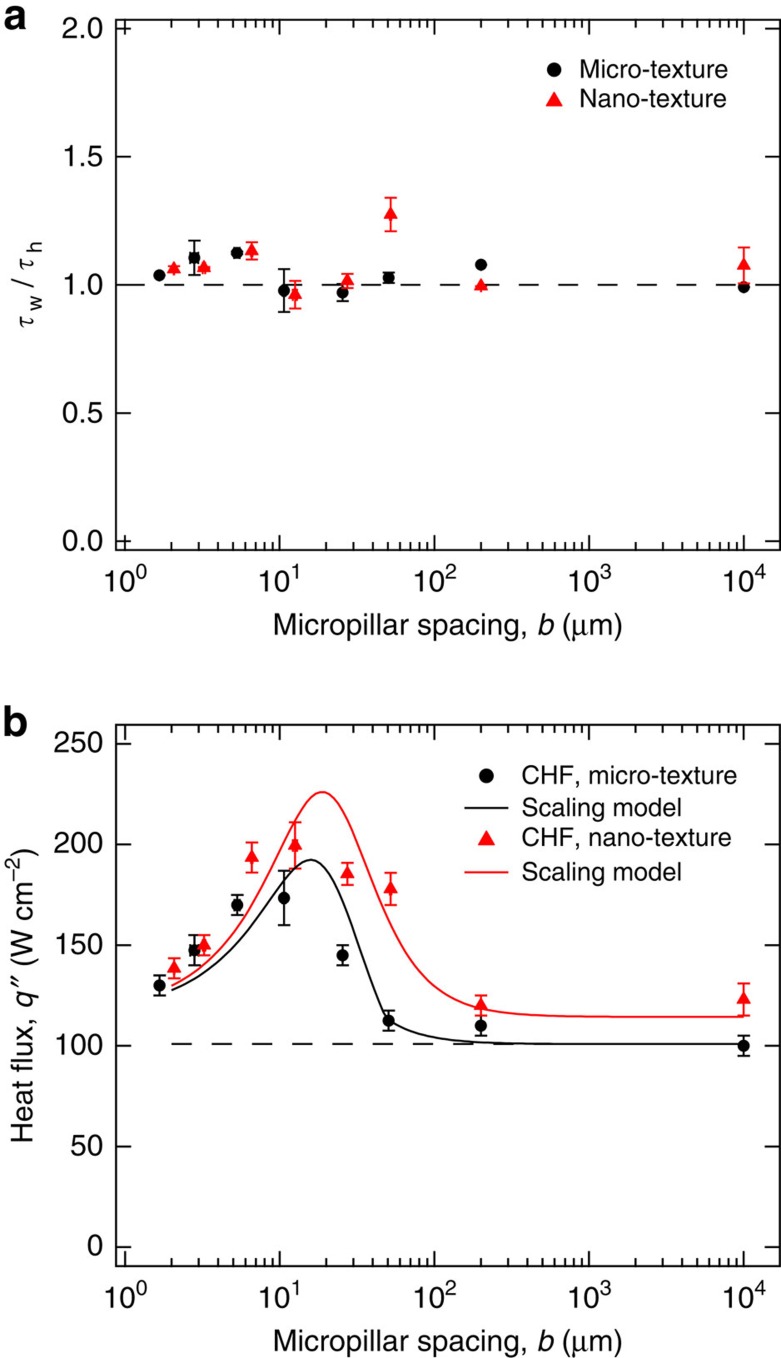
CHF scaling model results. (**a**) Plot of the ratio of calculated rewetting (*τ*_w_) and heating (*τ*_h_) timescales at the experimentally observed CHF values for both the micro- and nano-textured surfaces versus measured micropillar spacing *b*. The actual measured values of *a*, *b* and *h* have been used for all the samples (Table 1). The measured contact angle of water on silicon is *θ*∼30° (see Methods and Supplementary Fig. 5) and *T*_crit_–*T*_o_∼12 °C. (**b**) Plot of experimental CHF data and theoretical curves obtained using the CHF scaling model versus micropillar spacing *b* for the micro- and nano-textured surfaces. Average micropillar width of *a=*10 μm and height of *h=*12.75 μm were used for generating the theoretical CHF curves.

To verify the applicability of the scaling model and check its accuracy, we compare the calculated rewetting (*τ*_w_) and heating (*τ*_h_) timescales at the experimentally observed CHF values for all the micro- and nano-textured boiling samples in Fig. 7a. The ratio of *τ*_w_ and *τ*_h_ is plotted against the micropillar spacing *b* using experimentally measured values of micropillar width (*a*), height (*h*) and spacing for each sample (Table 1). Recall that the underlying hypothesis of the model was that CHF is encountered as soon as the rewetting timescale exceeds the heating timescale, which is borne out by the fact that most of the CHF data points in Fig. 7a fall on the horizontal line *τ*_w_/*τ*_h_=1.

Figure 7b plots the CHF curves obtained from equation (9) for both the micro-textured and nano-textured surfaces versus the micropillar spacing *b*, and compares them with the experimental CHF data. The CHF model nicely captures the maxima observed in the experimental CHF data and most of the CHF data points fall on the model curves within the margin of error. At large micropillar spacings (*b*≥200 μm), the rewetting of the dry spot is purely gravity-induced because either the liquid does not imbibe into the micropillars (*P*_c_<0 for micro-textured samples) or the imbibition liquid-front velocity *v*_i_ is smaller than the velocity of the sloshing liquid front *v*_g_ (nano-textured samples). In this regime, the slightly higher CHF for the perfectly wetting (*θ*_0_=0) nano-textured surface is explained by the non-zero sloshing-liquid pressure reduction term (Δ*P*_r_) for the partially wetting micro-textured surface (*θ*_0_∼30°). From *b*∼200 μm to *b*∼50 μm, the CHF increases for both the surfaces, albeit due to different reasons. The smaller increase for the micro-textured surface is due to a reduction in Δ*P*_r_ (decreasing *θ*_0_), whereas the larger increase for the nano-textured surface is because of imbibition-induced rewetting of the dry spot becoming active (*v*_i_>*v*_g_). For *b*<50 μm, imbibition-induced rewetting becomes active for both the surfaces with the CHF enhancement larger for the nano-textured surface because of a smaller micro-texture level liquid–solid contact angle *θ*_1_. Below a micropillar spacing of ∼10–20 μm, CHF for both surfaces starts to decrease with further reductions in *b*. This is because reducing *b* now increases the viscous pressure drop *P*_v_ to a larger degree than the imbibition capillary pressure *P*_c_, resulting in a reduced imbibition liquid-front velocity *v*_*i*_. For small micropillar spacings (*b*<<*a* and *b*<<*h*), the capillary pressure scales as *P*_c_*∼1/b*, whereas the viscous pressure drop scales as *P*_v_*∼1/b*^*2*^ (or *K*_v_∼*b*^2^).

## Discussion

We can summarize the role of the micropillars and the nanograss (or nanopillars) in CHF enhancement as follows: (i) For a partially wetting substrate (*θ*>0), both the micropillars and nanopillars enhance CHF to a small degree by decreasing the surface-level liquid–solid contact angle *θ*_0_, which enhances gravity-induced rewetting of a dry spot. (ii) The micropillars (but not the nanopillars) also lead to an additional larger enhancement in CHF because of the supplementary role played by the micro-imbibition layer in rewetting of the dry spot. A nano-imbibition layer is not feasible because of the comparatively lower rate of imbibition. (iii) For *θ*>0, the presence of nanopillars on top of the micropillars enhances CHF further by decreasing the micro-texture level liquid–solid contact angle *θ*_1_, which enhances the micro-imbibition layer-induced rewetting of the dry spot.

**Figure 8:**
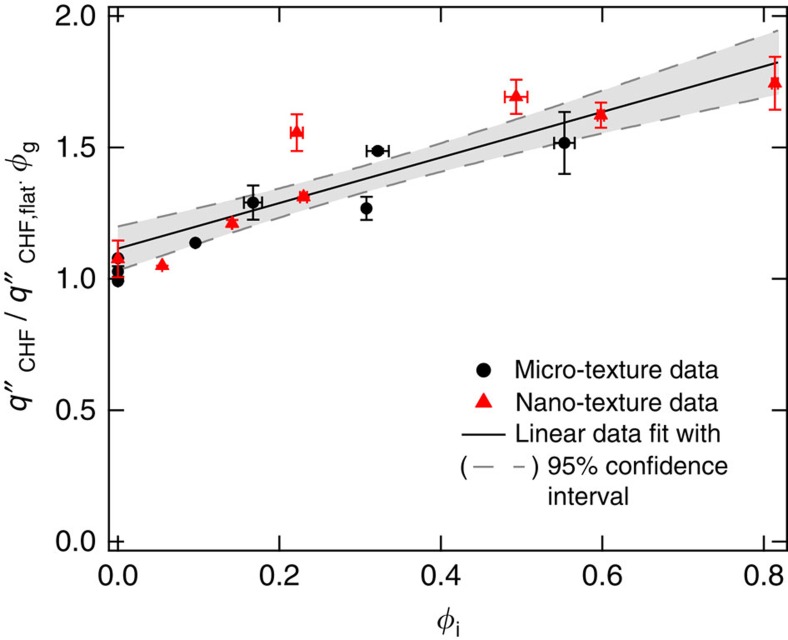
Effect of texture on CHF. The phenomenon of surface texture-induced CHF enhancement can be described using two non-dimensional parameters that correspond to the two distinct dry spot rewetting mechanisms. The gravity-induced enhancement parameter *φ*_g_=(1–*τ*_r_)/(1–*τ*_r,flat_) normalizes the effect of nanograss for non-imbibing textures, whereas the imbibition-induced enhancement parameter 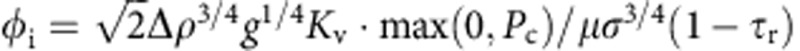 captures the effect of imbibition on CHF because of both the micro-texture and the nanograss. The approximately linear dependence of the non-dimensional quantity 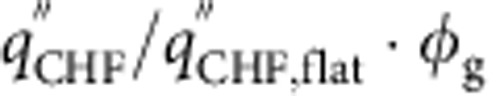 on *φ*_i_, illustrated by the plot, verifies the CHF scaling model (equation (10)). Here, 
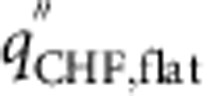
 is the CHF of flat silicon and the values of *φ*_g_ and *φ*_i_ were calculated using the measured values of *a*, *b* and *h* for all the samples (Table 1).

As there are two distinct mechanisms (gravity and imbibition-induced surface rewetting) by which texture influences CHF, we can identify two non-dimensional surface texture parameters that dictate CHF enhancement. Normalizing the expression of CHF obtained in equation (9) by the CHF of flat (non-imbibing) substrate 
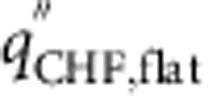
, we obtain


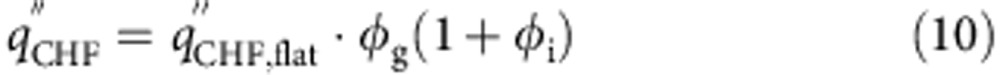


where *φ*_g_=(1–*τ*_r_)/(1–*τ*_r,flat_) is the gravity-induced and 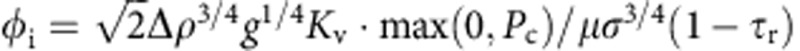 is the imbibition-induced non-dimensional surface texture CHF enhancement parameter. Whereas *φ*_g_ normalizes the CHF enhancement effect of nanograss at larger micropillar spacings, *φ*_i_ accounts for the imbibition-induced enhancement because of texture at both length scales. In Fig. 8, we further verify our CHF model by illustrating the linear dependence between the experimentally derived non-dimensional quantities 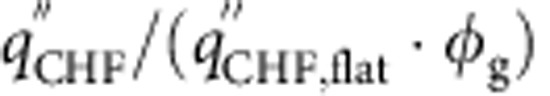 and *φ*_i_ as predicted by equation (10). Although we have experimentally verified the scaling CHF model using parametrically designed square micropillar surfaces, the surface texture-dependent terms *τ*_r_, *K*_v_ and *P*_c_ can be calculated or experimentally determined for other surface micro-texture morphologies.

In addition to correctly predicting the effect of surface texture on the boiling crisis, the coupled thermal-hydraulic formulation of the CHF model (equation (9)) also explains the experimentally observed^36^ reduction in CHF, in proportion to the heat capacity per unit area (*ρ*_s_*C*_s_*t*_s_), below a minimum value of the boiling substrate thickness *t*_s_. The expression for the heating timescale developed in equation (4) relies on the substrate thickness *t*_s_ (∼0.6 mm) being less than the characteristic thermal diffusion length 
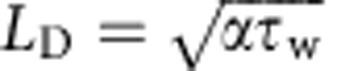
 (∼0.6–0.8 mm). For *t*_s_>*L*_D_, the heating of the substrate during the dry spot rewetting phase should be limited to a depth *L*_D_, making CHF independent of *t*_s_ for thicker substrates^36^. A more general expression for CHF can therefore be given as


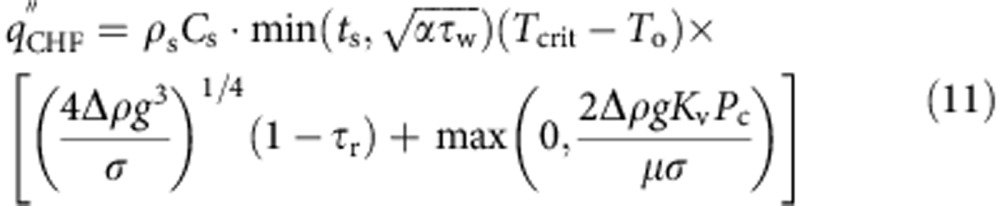


In summary, we show the existence of maxima in CHF enhancement at intermediate texture density for pool boiling on thin (∼0.6 mm) micro- and nano-textured surfaces. Using liquid imbibition experiments and quantitative infrared temperature measurements of dry spots on the boiling surface, we show direct evidence that the boiling crisis is dictated by characteristic dry spot heating and rewetting timescales. Based on these observations, we develop a coupled thermal-hydraulic model for CHF, given by equation (9), which shows how surface textures with an optimum design for liquid imbibition can maximize the value of CHF. Moreover, the model explains why completely wetting (*θ*_0_∼0) but non-imbibing surfaces have only a slightly higher CHF than flat partially wetting or hydrophobic surfaces^20^. Combined with the concept of thermal diffusion length 
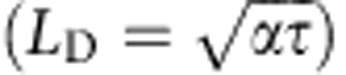
, the model also elucidates why CHF decreases linearly with substrate thickness below a minimum thickness value^36^. These new insights about surface texture-induced CHF enhancement and the phenomena of boiling crisis in general can guide the development of new techniques for enhancing CHF even further than is currently possible. This has important implications for a broad swathe of industries that use boilers and boiling heat exchangers in terms of reduced capital investment, enhanced energy efficiencies and reductions in greenhouse gas emissions.

## Methods

### Fabrication of textured boiling surfaces

Using deep reactive ion etching and employing patterned photoresist as an etch mask, square micropillars were etched on the top surface of a 650-μm-thick double-sided polished <100> silicon wafer. After removing the photoresist and cleaning the wafer in Piranha solution, nanograss was etched on top of the micropillars (for the nano-textured samples only) using a custom reactive ion etch recipe. The wafer was then cleaned again in Piranha solution and a 1 × 2-cm^2^ thin-film heater was patterned on its backside by depositing a 150-nm Ti layer (heater) followed by a 300-nm Ag layer (contact pads) using e-beam evaporation and shadow masking. The wafers were diced into 5 × 5 cm^2^ samples using photoresist to protect the top surface from contamination. Photoresist was stripped off and samples were cleaned in acetone and isopropanol. Just before the boiling experiments, the samples were again cleaned in acetone, isopropanol and DI water to remove any organic contaminants.

### Surface characterization

The height, width and spacing of the micropillars was measured using scanning electron microscopy and the values for all the samples are given in Table 1. The morphology of nanograss on the surface of micropillars was characterized using scanning electron microscopy imaging, whereas an average nanograss roughness value of *r*_ng_=3.43 was determined using atomic force microscopy measurements conducted on nanograss grown on a flat silicon substrate (Supplementary Fig. 2). The morphology of the nanograss at different locations of the micropillar surfaces was found to be similar. Further, it is important to note that above a critical roughness of 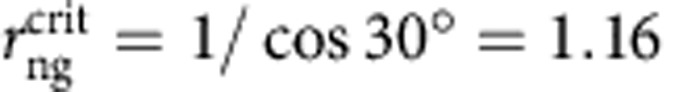, the micro-texture-level contact angle *θ*_1_ becomes zero. Therefore, variation of nanograss roughness on the boiling surface is of no consequence as long as 
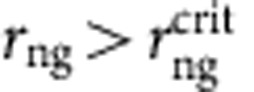
. To determine the inherent silicon–water contact angle (*θ*) on the boiling samples, we need to account for the presence of a native oxide layer formed due to both oxidation in air^52^ (*θ*∼36°) and Piranha cleaning^53^ (*θ*∼22°). The contact angle of DI water on a flat silicon wafer with native oxide, and cleaned in the same manner as the boiling samples, was measured to be *θ*∼30° (Supplementary Fig. 5).

### CHF measurements

DI water used for boiling experiments was degassed by boiling for 20 min in a microwave. It was then poured into the stainless steel boiling chamber containing the textured-silicon boiling sample. To keep the DI water at saturation, the inner walls of the boiling chamber were maintained at 100 °C by flowing a hot 1:1 solution of water and propylene glycol in a metal jacket surrounding the chamber. The high-speed optical camera was focused on the boiling surface at an angle of 16° with the horizontal, whereas the infrared camera was focused on the Ti thin-film on the backside of the boiling sample using a gold mirror. The DI water was boiled for ∼1 h on the textured sample to completely degas the system. Power to the substrate was switched off for ∼1/2 h and then the applied heat flux was increased in steps of 10 W cm^−2^ at 5 min intervals until a CHF event was encountered. In many instances, the heat flux increments were reduced to 5 W cm^−2^ close to an expected CHF event to reduce the error in CHF measurements. CHF was assumed to occur when the Ti heater underwent a rapid rise in temperature, which fractured the heater in most cases. Two nominally identical samples were tested for each type of textured boiling surface.

## Additional information

**How to cite this article:** Dhillon, N. S. *et al*. Critical heat flux maxima during boiling crisis on textured surfaces. *Nat. Commun.* 6:8247 doi: 10.1038/ncomms9247 (2015).

## Supplementary Material

Supplementary Information Supplementary Figures 1-5, Supplementary Notes 1-5 and Supplementary References

Supplementary Movie 1 Raw infrared videos illustrating a CHF event on the nano-textured surface with 2 μm-spaced micropillars. Playback speed is 1/50 x real-time.

Supplementary Movie 2 Imbibition of 10 cSt silicone oil into the micro-textured surfaces with different micropillar spacing. Playback speed is 1x real-time.

## Figures and Tables

**Table 1:** Critical heat flux measurements on micro- and nano-textured surfaces.

**Sample type**	***b***_**d**_ **(μm)**	***b*****(μm)**	***h*****(μm)**	**CHF (W** **cm**^**−2**^)
Micro	2	1.7	15.6	130
Micro	3	3, 2.6	17.1, 16.4	140, 155
Micro	5	5.5, 5.1	12.2, 12.1	171, 169
Micro	10	10.9, 10.4	11.2, 10.7	160, 187
Micro	25	25.6, 25.4	10.3, 10.2	150, 140
Micro	50	50.6, 50.6	10.2, 9.6	115, 110
Micro	200	200	15.8	110
Micro	—	—	—	100,100
Nano	2	2.1, 2.1	16.4, 15.7	140, 137
Nano	3	3.2, 3.3	16.9, 16.0	150, 150
Nano	5	6.7, 6.5	13.3, 10.4	201, 186
Nano	10	12.2, 13	11.9, 11.2	211, 188
Nano	25	27.4, 27.4	10.8, 10.8	191, 180
Nano	50	52.6, 51.5	10.7, 9.6	170, 186
Nano	200	200	15.7	120
Nano	—	—	—	131, 115

CHF, critical heat flux.

*b*_d_ is design micropillar spacing, *b* is measured micropillar spacing, *h* is measured micropillar height and CHF is measured critical heat flux. Measurements on the two nominally similar samples are listed in order.
