# Supplementary material for: Critical heat flux maxima during boiling crisis on textured surfaces
Source: Nat Commun. 2015 Sep 8;6:8247. doi: 10.1038/ncomms9247 (PMC4569857; doi:10.1038/ncomms9247)
Supplement: Supplementary Information — Supplementary Figures 1-5, Supplementary Notes 1-5 and Supplementary References [file ncomms9247-s1.pdf]

Supplementary Figures

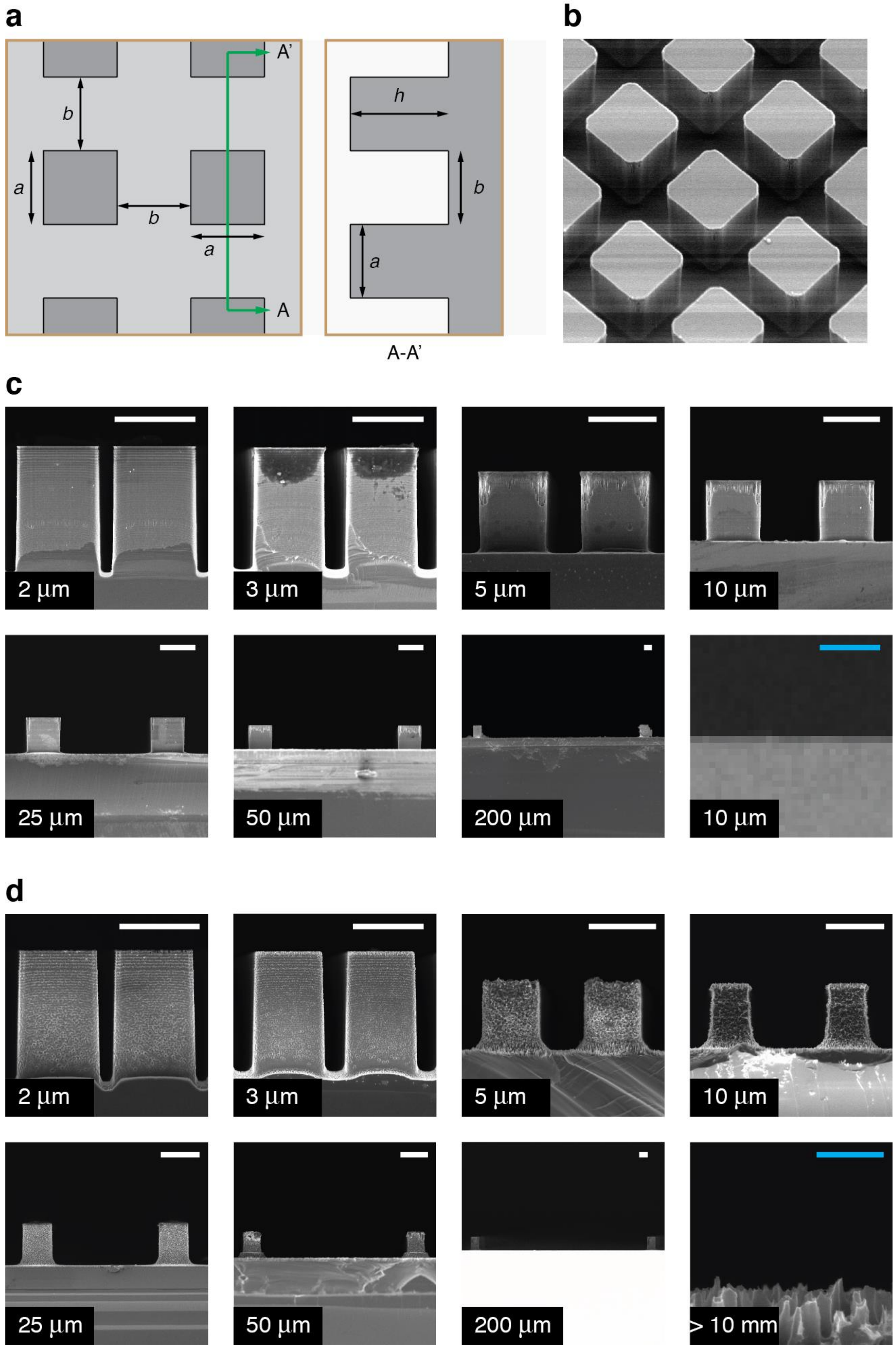

Supplementary Figure 1 | Micro and nano-textured boiling surfaces. (**a**) A schematic of the textured boiling surfaces. (**b**) An isometric view of the square array of square micropillars. (**c**) The micro-textured surface consists of a square array of plain square micropillars of width a ~ 10 µm. Cross sectional SEM images of the micropillars are shown here. To study the effect of texture on CHF, the spacing between the micropillars (b) is varied from ~2 µm to ~10 mm (flat surface). (**d**) The nano-textured surfaces have an approximately 10 nm length scale nano-texture called nanograss etched on top of the micropillars using an additional dry etching step. Blue scale bar 1 µm. White scale bar 10 µm. The average height of the micropillars is h ~ 12.75 µm.

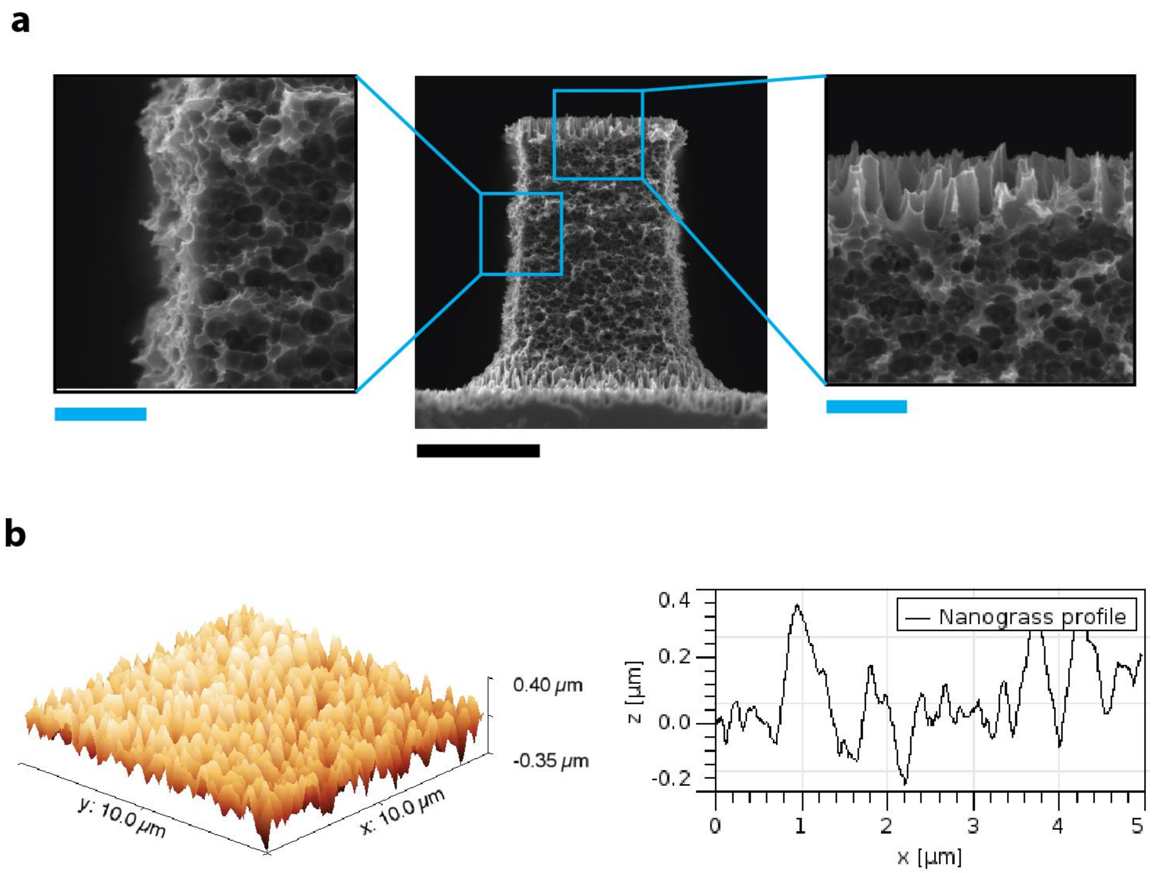

Supplementary Figure 2 | Nanograss areal surface roughness. (**a**) SEM images of the nanograss on the top and side of a micropillar. The design micropillar spacing in this case is 10 µm. It is clear that the nanograss covers the base as well as all exposed surfaces of the micropillar. Black scale bar 5 µm. Blue scale bar 1 µm. (**b**) A 3-D AFM image of the surface topology of nanograss grown on a flat silicon substrate. For the projected area of 100 µm^2, the total measured surface area was found to be 343 µm^2, which yields an areal surface roughness for the nanograss of r_{ng} = 3.43. Also shown is a 1-D profile of the nanograss.

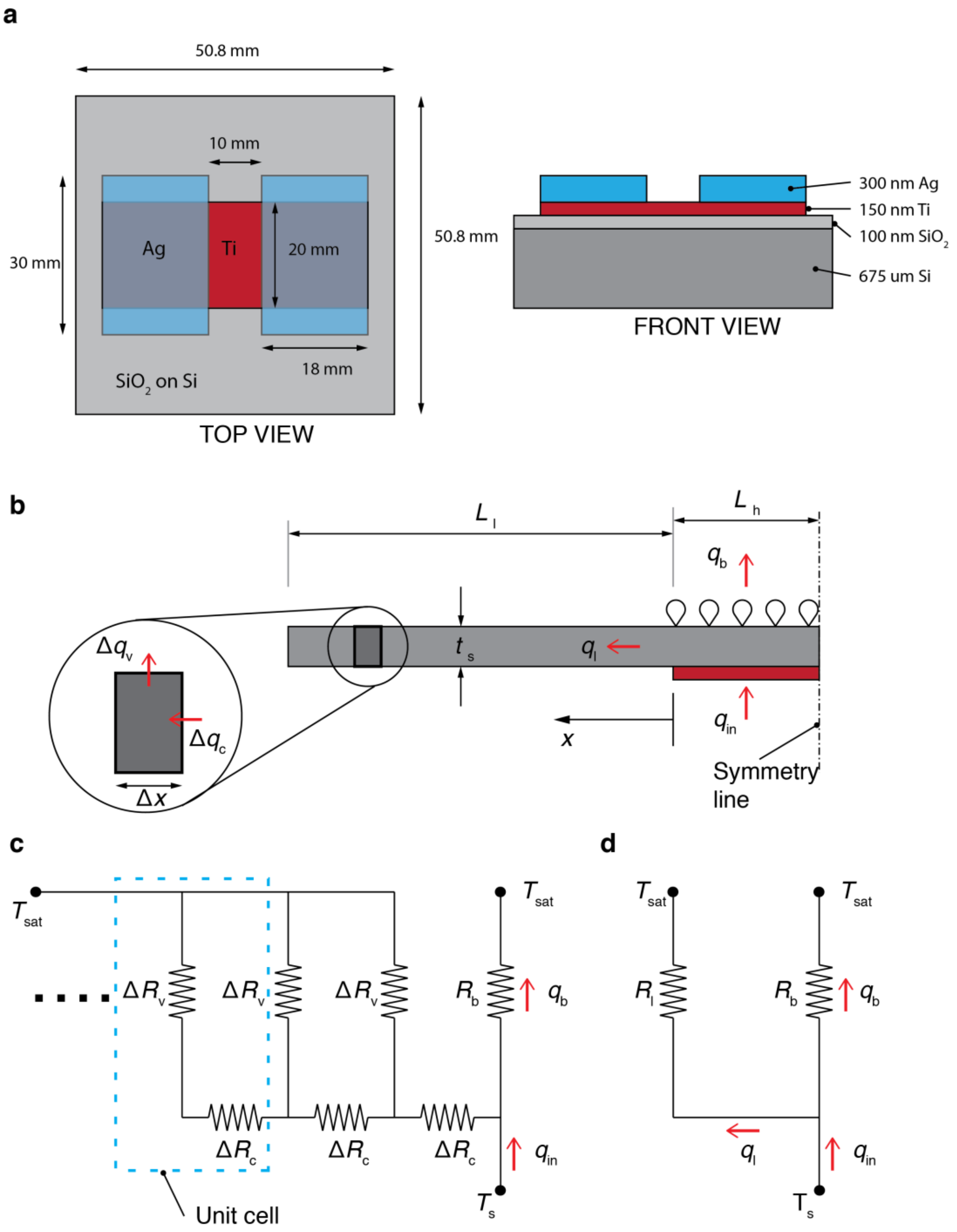

Supplementary Figure 3 | Heater and boiling substrate. (**a**) Dimensions of the boiling substrate and the patterned thin-film heater. (**b**) Heat flow modes in the boiling substrate represented as a 2-D domain problem. (**c**) The thermal resistance circuit that can model the heat flow via conduction and convection in the substrate. (**d**) A simplified thermal circuit obtained by combining all the discretized conduction and convection thermal resistances. In Supplementary Note 1, we show that the heat loss due to conduction is negligible: $q_l << q_{in}$ (or $q_b \approx q_{in}$).

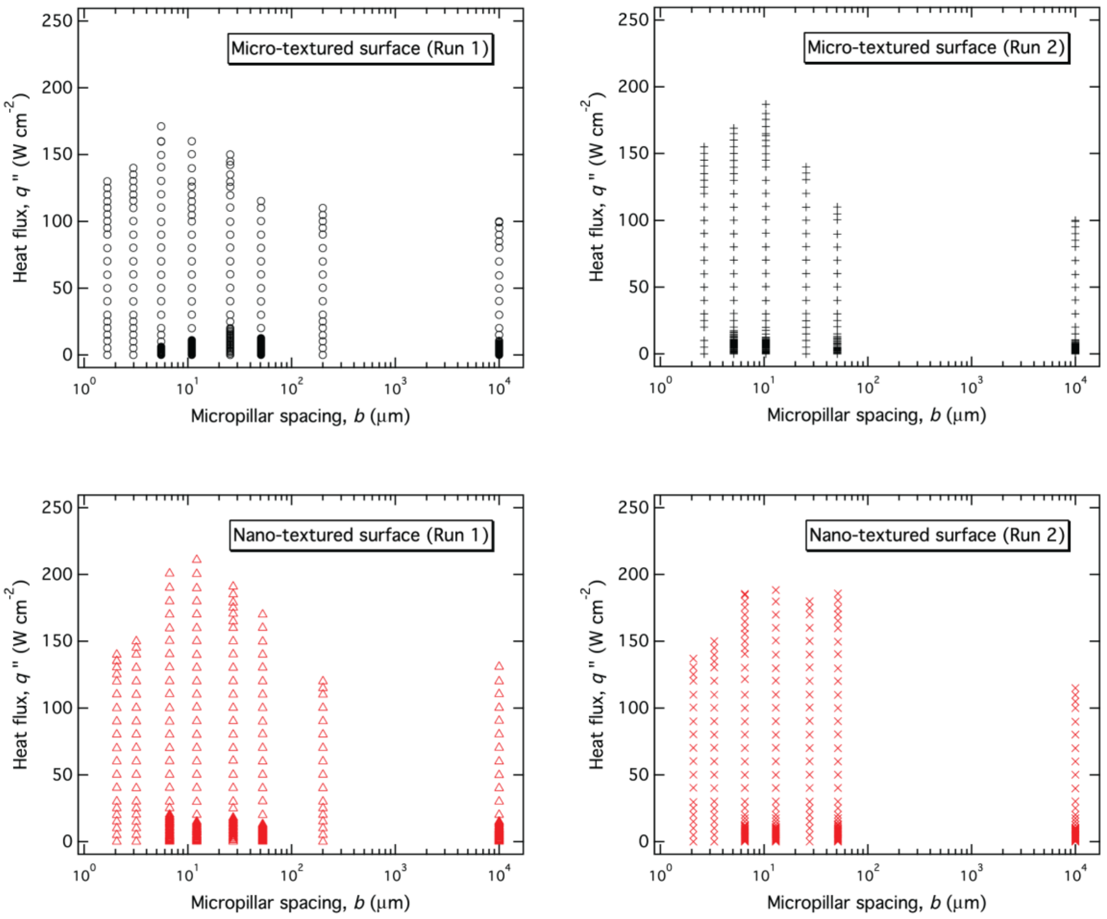

Supplementary Figure 4 | Heat flux increments applied to obtain CHF. For both the micro and nano-textured surfaces two nominally similar samples were tested. The graphs show the incrementally applied heat flux values until the sample encountered a CHF event.

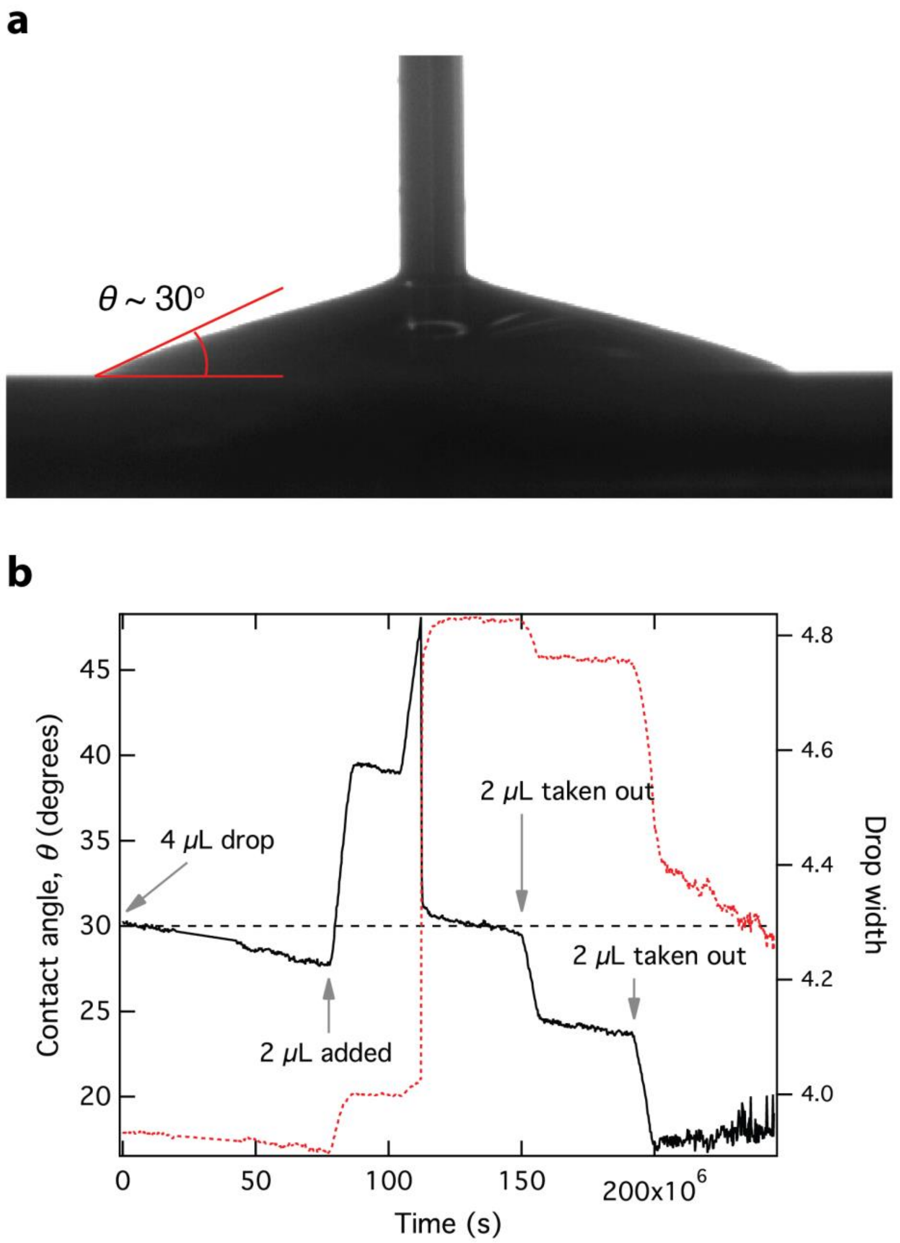

Supplementary Figure 5 | Contact angle of water on silicon. (**a**) Optical image of a needle-dispensed 4 µL drop of deionized water on plain silicon. Equilibrium contact angle of q ~ 30° is measured. (**b**) By adding and taking out water from the drop, the equilibrium contact angle is verified and the advancing (~40°) and receding (~24°) contact angles are also measured.

Supplementary Notes

Supplementary Note 1 – Hydraulic and thermal characteristics of the boiling substrate

The configuration and dimensions of the boiling substrate used in the pool boiling CHF experiments are shown in Supplementary Fig. 3. The 650 µm-thick silicon sample is 5 cm x 5 cm square and has a 100 nm electrically insulating SiO_2 layer deposited on its backside. The 1 cm long and 2 cm wide portion of the 150 nm-thick Ti thin film patterned on the backside that lies between the two 300 nm-thick silver contact pads is the thin-film heater, which heats the substrate. This precisely defines a 1 cm x 2 cm boiling area on the top surface of the substrate in contact with the liquid. This size of the boiling area was chosen to be large enough to ensure boiling behavior similar to that on an infinitely large boiling surface. It should be mentioned that for heaters that are too small, CHF increases above the normal value. However, Gogonin and Kutateladze[1] and Theofanous et al.[2] have experimentally shown that the CHF of a flat heater is equal to that of an infinitely large boiling surface as long as the minimum heater dimension is larger than $2\sqrt{\sigma / \Delta\rho g}$ (~5 mm for water). Note that the maximum diameter of the critical dry spot in our analysis ($2\lambda \sim 2\sqrt{\sigma / \Delta\rho g}$) is also equal to 5 mm, ensuring that the dry spot that leads to a CHF event is completely inside the heated area (1 cm x 2 cm).

Since only a portion (1 cm x 2 cm) of the 5 cm x 5 cm silicon substrate in contact with the liquid is heated, the accuracy of the pool boiling CHF measurements could possibly be affected by heat losses due to conduction away from the heated area. However, here we show that due to the small substrate thickness (t_s ~ 0.6 mm) and because the heat transfer coefficients associated with pool boiling are much larger than those associated with liquid convection, the conduction losses are within the margin of error of CHF measurements and can be neglected. Supplementary Fig. 3b illustrates the heat flow modes in the substrate using a 2-D representation, where q_{in} is the incoming heat flow rate per unit time (supplied by the Ti heater), q_b is the heat flow dissipated in boiling, and q_l is the heat lost by conduction away from the heated area. Since the mechanical supports at the edge of the sample are thermally insulating, the heat lost by conduction has to be dissipated via convective heat transfer to the liquid above. This 2-D

conjugate heat transfer problem can be solved by discretizing the solid domain along the x-direction (Δx = 1 mm), assuming 1-D conduction along x, and uniform liquid convective heat loss out of the discretized element. The problem can now be represented using the thermal resistance circuit shown in Supplementary Fig. 3c, where the unit cell corresponding to conduction/convection in the discretized domain unit is repeated to model the entire domain. The unit cell conduction resistance (per unit width out of plane) is given by ΔR_c = $\Delta x / k_\mathrm{s} t_\mathrm{s}$ = 0.015 Km/W, where k_s = 110 W/mK is the thermal conductivity (at ~100°C) and t_s = 0.6 mm the thickness of the silicon substrate. The convection resistance ΔR_v is given by ΔR_v = $1/h_\mathrm{v}\Delta x$, where h_v is the heat transfer coefficient for liquid natural convection. The following correlation[3] can be used to evaluate h_v:

$$\begin{gathered} \overline{Nu}_\mathrm{L} = 0.54\, Ra_\mathrm{L}^{1/4} \quad (10^4 \le Ra_\mathrm{L} \le 10^7) \\ Ra_\mathrm{L} = \frac{g\beta(T_\mathrm{s} - T_\infty)L^3}{\nu\alpha},\ L = \frac{A_\mathrm{s}}{P},\ Nu = \frac{hL}{k} \end{gathered} \tag{1}$$

Using water properties at 100°C and $T_\mathrm{s} - T_\infty$ ~ 10°C, we obtain h_v = 1.2×10^3 W $\mathrm{m}^{-2}\,\mathrm{K}^{-1}$ and ΔR_v = 0.83 K m W^{-1}. Using the method of series and parallel resistances, the repeating array of conduction/convection thermal resistance unit cell can be combined to get the net thermal loss resistance R_l (Supplementary Fig. 3d):

$$R_\mathrm{l} = R_{m=M}, \quad R_m = \left(\frac{1}{\Delta R_\mathrm{v}} + \frac{1}{\Delta R_\mathrm{c} + R_{m-1}} \right), \quad R_{m=1} = \Delta R_\mathrm{v} \tag{2}$$

where M is the number of discretized domain units. Using an average unheated substrate length of L_l = $(20+15)/2$ = 17.5 mm (due to asymmetric heater dimensions), we get M = $L_\mathrm{l} / \Delta x$ ~ 17. This yields a thermal loss resistance of R_l = 0.106 K m W^{-1}.

The boiling thermal resistance is given by $R_\mathrm{b} = 1/h_\mathrm{b}L_\mathrm{h}$, where L_b = (5+10)/2 = 7.5 mm is the average heated length and h_b is the boiling heat transfer coefficient. Since h_b ~ $q'' / (T_\mathrm{s} - T_\mathrm{sat})$ and $T_\mathrm{s} - T_\mathrm{sat}$ is observed to be approximately 20°C for all samples (100 W cm^{-2} $\le q''_\mathrm{CHF} \le$ 200 W cm^{-2}), a conservative value of h_b ~ 5×10^4 W $\mathrm{m}^{-2}\,\mathrm{K}^{-1}$ can be assumed, which yields a boiling thermal resistance of R_b = 0.0027 K m W^{-1}. The fractional heat loss by conduction can now be calculated using $q_\mathrm{l} / q_\mathrm{in}$ ~ $R_\mathrm{b} / (R_\mathrm{b} + R_\mathrm{l})$ to get $q_\mathrm{l} / q_\mathrm{in}$ = 2.5 %. Note that this is a conservative estimate given that parts of the

substrate are covered by thermally insulating Teflon clamps, and because we calculated h_b at q''_{CHF} = 100 W cm^{-2} (which yields the lowest value of h_b). This means that the conductive heat loss should lead to a CHF measurement error of < 2.5 W cm^{-2} for all the samples regardless of the measured CHF value, and further the error should be systematic (of the same sign and approximately the same value). For these reasons, and because the error is well within the inherent CHF data acquisition error of 10 W cm^{-2}, we can ignore these losses in our analysis.

Supplementary Note 2 – Experimental uncertainty analysis

The objective of the CHF experiments is to measure the changes in CHF on a textured surface due to the change in spacing *b* between the plain and nanograss micropillars that give the surface its texture. Since the CHF is obtained by gradually increasing the applied heat flux in maximum 10 W cm^{-2} increments, the uncertainty in CHF measurement should be approximately 10 W cm^{-2}. However, it should also be kept in mind that CHF measurements are not exactly repeatable due to the inherent statistical nature of the boiling crisis phenomenon and its dependence on experimental conditions such as sample cleanliness, water purity etc. We therefore measured the CHF values for two nominally similar samples for each type of textured surface studied (see Table 1). The uncertainty in CHF (vertical error bars in Figs. 2, 7, and 8) is taken to be equal to the difference of these two measurements or 10 W cm^{-2}, whichever is greater.

Although the spacing between the micropillars *b* can be determined quite accurately using SEM imaging, it varied slightly between the two nominally similar samples due to the inherent variation in the conditions encountered in microfabrication processes such as photolithography and deep reactive ion etching (DRIE). For plotting the CHF values versus micropillar spacing *b* in Figs. 2 and 7, we used the average value of *b* obtained from the two nominally similar samples and denoted the difference as the uncertainty in *b* using the horizontal error bar (not visible if smaller than the data point icon). For the plot in Fig. 8, the variable f_{i} was calculated using the respective measured geometrical dimensions of the two nominally similar samples (Table 1). The mean value and experimental uncertainty (horizontal error bar) of f_{i} were calculated using the same approach as described above for *b*.

The experimentally determined heating timescales (t_h) plotted in Fig. 6d are based on infrared measurements of hot spots with the maximum observed temperature ramp rates for each sample, corresponding to $T_{\mathrm{crit}} - T_{\mathrm{o}} \sim 12^{\circ}\mathrm{C}$. An extremely small temperature measurement error of $DT^{\mathrm{e}} \sim \pm 20$ mK for the infrared camera means that the maximum possible error in the experimentally determined heating timescale ($Dt_{\mathrm{h}}^{\mathrm{e}}$) for an

analyzed hot dry spot is quite small and can be neglected: $\Delta t_{\mathrm{h}}^{\mathrm{e}} / t_{\mathrm{h}} \sim 8\Delta T^{\mathrm{e}} / (T_{\mathrm{crit}} - T_{\mathrm{o}}) =$ 0.67%.

Supplementary Note 3 – Comparison of CHF data with the liquid wicking correlation[4]

Rahman et al.[4] have proposed a correlation to explain surface texture-induced CHF enhancement based purely on wicking of liquid into the surface textures. The CHF of a wicking surface is given by

$$q''_{\text{CHF, w}} = q''_{\text{CHF, Zuber}}(1 + Wi) \tag{3}$$

where $q''_{\text{CHF, Zuber}}$ is the expression for CHF obtained using conventional hydrodynamic theory, first proposed by Zuber[5], that does not account for surface effects:

$$q''_{\text{CHF, Zuber}} = 0.131 r_{\text{v}}^{1/2}\left[s g\left(r_{\text{l}} - r_{\text{v}}\right)\right]^{1/4} \tag{4}$$

The wicking number Wi is given by

$$Wi = \frac{\dot{V}''_{\text{o}}\rho_{\text{l}}}{\rho_{\text{v}}^{1/2}\left[\sigma g\left(\rho_{\text{l}} - \rho_{\text{v}}\right)\right]^{1/4}} \tag{5}$$

where the wicked volume flux $\dot{V}''_{\text{o}}$ is measured by tracking the flow of liquid from a capillary tube onto the textured surface:

$$\dot{V}''_{\text{o}} = \frac{1}{A_{\text{w}}}\left(\frac{dV}{dt}\right)_{t=0} \tag{6}$$

Here A_{w} is the initial wetting area of the liquid from the capillary tube that wets the textured surface, and $\left(dV/dt\right)_{t=0}$ is the volume flow rate measured at time $t = 0$ after the liquid has just touched the surface.

Since Rahman et al. relied purely on experimental measurements to calculate $\dot{V}''_{\text{o}}$, we will need to develop a theoretical expression for the same to compare our CHF data to this correlation. Consider a circular patch of bulk liquid of radius R_{w} imbibing radially into the square micropillar surface. The radial viscous pressure drop dP_{v} is given by

$$\frac{dP_{\text{v}}}{dr} = \frac{m v}{K_{\text{v}}} \tag{7}$$

where m is the dynamic viscosity and v the radial velocity of the liquid, and K_{v} is the permeability of the texture (see equation (2)). Using conservation of mass, the velocity v at radius r can be expressed in terms of the velocity v_{i} of the liquid imbibition front at

$r = R$: $v = v_i \cdot R / r$. Substituting this into Supplementary equation (7) and integrating from $r = R_w$ to $r = R$, we get

$$\Delta P_v = \frac{\mu v_i}{K_v} R \ln\left(\frac{R}{R_w}\right) \tag{8}$$

Since the total viscous pressure drop ΔP_v has to be equal to the capillary pressure P_c (given by equation (1)) at the imbibition front and because the imbibition front velocity can be expressed as $v_i = dR/dt$, we can write this as

$$\frac{P_c K_v}{\mu} dt = R \ln\left(\frac{R}{R_w}\right) dR \tag{9}$$

Integrating this equation, we can calculate the time Δt required for the liquid imbibition front to travel from $R = R_w$ to $R = R_w + \Delta R$:

$$\frac{P_c K_v}{\mu} \Delta t = \frac{\left(R_w + \Delta R\right)^2}{4}\left[2\ln\left(1 + \frac{\Delta R}{R_w}\right) - 1\right] + \frac{R_w^2}{4} \tag{10}$$

The volume of the liquid imbibed into the texture is given by

$$\Delta V = 2\pi R_w \Delta R \left[\frac{(a+b)^2 - a^2}{(a+b)^2}\right] h \tag{11}$$

where a is the width, h the height, and b the spacing of the square array of square micropillars. The wicked volume flux $\dot{V}_o''$ can now be calculated using Supplementary equations (10) and (11) to get:

$$\dot{V}_o'' = \frac{1}{\pi R_w^2}\frac{\Delta V}{\Delta t} = \frac{8\Delta R\left(b + 2a\right) bh \cdot P_c K_v}{R_w \left(a+b\right)^2 \mu\left\{\left(R_w + \Delta R\right)^2\left[2\ln\left(1 + \Delta R / R_w\right) - 1\right] + R_w^2\right\}} \tag{12}$$

In their supplementary information, Rahman et al. mention that the diameter of the wetted area D_w was measured to be between 1.3 and 1.6 mm. We therefore take R_w = $D_{avg}/2$ = 0.725 mm. However, contrary to Rahman et al.'s assertion that $\dot{V}_o''$ is a phenomenological parameter that characterizes the inherent wickability of the surface, we find that $\dot{V}_o''$ depends both on R_w and ΔR. To compare our CHF data with this wicking correlation, we assume a value of ΔR = 2.5 mm that yields the same maximum CHF as the experimental data. Plotting Supplementary equation (3) in Fig. 2 using $q''_{\text{CHF, Zuber}} = 100\ \text{W cm}^{-2}$, we find that the wicking correlation does not agree with the experimental data for $b \le 10\ \mu\text{m}$.

Supplementary Note 4 – Derivation of imbibition capillary pressure and surface texture permeability

Capillary pressure (P_c) is the jump in pressure encountered across curved liquid-vapor interfaces due to interfacial surface tension. For surfaces with a uniform radius of curvature P_c can be easily calculated using a force balance approach. However, because of the non-uniform curvature (both in space and time) of the advancing liquid front of the micro-imbibition layer, we use a work-energy approach. Work done by the capillary pressure in moving forward a liquid volume dV across the interface: $dW = P_c \cdot dV$. Net change in energy of the system due to surface wetting by the liquid = dE. By conservation of energy $dW + dE = 0 \Rightarrow P_c = -dE/dV = -\Delta E/\Delta V$. Considering the wetting of a unit cell (Fig. 5a) of volume $\Delta V = h[(a+b)^2 - a^2]$ and neglecting wetting of the micropillar tops, we can calculate the net change in system energy by accounting for the loss of the existing solid-vapor interface ($\Delta E_1 = -[(a+b)^2 - a^2 + 4ah]r_{ng}\sigma_{sv}$) and the creation of new solid-liquid ($\Delta E_2 = [(a+b)^2 - a^2 + 4ah]r_{ng}\sigma_{sl}$) and liquid-vapor ($\Delta E_3 = [(a+b)^2 - a^2]\sigma_{lv}$) interfaces: $\Delta E = \Delta E_1 + \Delta E_2 + \Delta E_3 = [b(2a+b) + 4ah]r_{ng}\left(\sigma_{sl} - \sigma_{sv}\right) + b(2a+b)\sigma_{lv}$. Using the Laplace equation[6], we can write $r_{ng}(\sigma_{sl} - \sigma_{sv}) = -r_{ng}\sigma_{lv}\cos\theta = -\sigma_{lv}[\cos\theta_1]$, where $\theta_1 = \cos^{-1}[\min(1, r_{ng}\cos\theta)]$ is the apparent contact angle at the micro-texture level. Note that in doing so we have limited to σ_{lv} the maximum contribution of the term $r_{ng}(\sigma_{sv} - \sigma_{sl})$ in the original expression for ΔE. This is because the capillary force imbibing the liquid between the micropillars maximizes at $\theta_1 = 0$. Substituting this into ΔE, we get the following expression for the capillary pressure: $P_c = -\Delta E/\Delta V = \sigma_{lv}[4a\cos\theta_1 / b(2a+b) - (1-\cos\theta_1)/h]$.

To develop a scaling expression for permeability of surface with a square array of square micropillars, we consider two extreme scenarios. For large micropillar spacings (b >> 1), the flow can be treated as free surface flow on a flat plate yielding the following relation between the pressure gradient ∇P and the mean flow velocity v_m: $\nabla P \sim 3\mu v_m / h^2$. At small micropillar spacings (b << 1), the flow between the micropillars can

be treated as flow between parallel plates, but with the average velocity reduced by a factor of ~1/2 to account for flow retardation by the horizontal surface: $\nabla P \sim 24 m v_{\mathrm{m}} / b^2$. At intermediate micropillar spacings, we can combine these results while noting that the parallel plate flow resistance component is only active along a fractional length $a/(a+b)$ of the unit cell: $\nabla P \sim m v_{\mathrm{m}} [\, 3/h^2 + 24a/b^2(a+b)\,]$. This yields a surface texture permeability of $K_{\mathrm{v}} = [\, 3/h^2 + 24a/b^2(a+b)]^{-1}$ where $\nabla P \sim m v_{\mathrm{m}} / K_{\mathrm{v}}$.

Supplementary Note 5 – Extrapolation of silicone oil imbibition results to water

To verify the scaling imbibition model and provide experimental validation of the theoretically calculated dry spot rewetting timescales, imbibition experiments were conducted using silicon micro-textured surface samples. Although DI water was used to obtain the pool boiling CHF data, it was found to be unsuitable for performing the imbibition experiments due to its relatively high vapor pressure and low liquid viscosity (n) at room temperature. Under the intense lighting conditions used for the high-speed optical imaging, necessitated by the relatively fast imbibition velocity v_{i} ($\sim 1/n$), a substantial amount of evaporation can occur from the thin water film imbibing into the micro-textures. Further, the low viscosity of water leads to inertial effects when the imbibition samples touches the liquid pool. The contact line of the bulk liquid oscillates up and down the sample surface introducing errors into the measurements. To avoid these problems and to achieve better experimental accuracy, 10 cSt silicone oil, a liquid with substantially lower vapor pressure and the preferred choice in literature for surface tension studies, was instead employed for the imbibition experiments.

The scaling imbibition model developed in this paper and verified using silicone oil imbibition experiments depends, for a given texture morphology, only on the liquid surface tension (s_{lv}), liquid viscosity (n) and the liquid-solid contact angle (q). Provided the values of these three parameters are accurately known, it is easy to extrapolate the results of silicone oil imbibition experiments to other liquids including water. The ratio of the liquid imbibition times for water ($t_{\mathrm{i}}^{\mathrm{water}}$) and silicone oil ($t_{\mathrm{i}}^{\mathrm{oil}}$) can be expressed as

$$\frac{t_{\mathrm{i}}^{\mathrm{water}}}{t_{\mathrm{i}}^{\mathrm{oil}}} = \left(\frac{m^{\mathrm{water}}}{m^{\mathrm{oil}}} \cdot \frac{s_{\mathrm{lv}}^{\mathrm{water}}}{s_{\mathrm{lv}}^{\mathrm{oil}}} \right) \left(\frac{G^{\mathrm{oil}}}{G^{\mathrm{water}}} \right) \tag{13}$$

where $G = [4a/b(2a+b)\cos q_1 - (1-\cos q_1)/h]$ is a parameter that depends on the morphology of the square micropillar array and the apparent contact angle at the micro-texture level $q_1 = \cos^{-1}[\min(1, r_{\mathrm{ng}} \cos q)]$.

This extrapolated experimental imbibition time for water ($t_{\mathrm{i}}^{\mathrm{water}}$ or $t_{\mathrm{w,\,i}}^{\mathrm{water}}$) can now be combined with the gravity induced rewetting timescale $t_{\mathrm{w,\,g}}^{\mathrm{water}}$ (equation (5)) to obtain the experimentally measured dry spot rewetting timescale using equation (8)

$$t_{\mathrm{w}}^{\mathrm{water}} = \left[1/t_{\mathrm{w,\,g}}^{\mathrm{water}} + \max\left(0,\, 1/t_{\mathrm{w,\,i}}^{\mathrm{water}}\right) \right]^{-1} \qquad (14)$$

Supplementary References

1. Gogonin, I. I. & Kutateladze, S. S. Critical heat flux as a function of heater size for a liquid boiling in a large enclosure. *J. Eng. Phys.* **33,** 1286–1289 (1977).
2. Theofanous, T. G., Tu, J. P., Dinh, A. T. & Dinh, T. N. The boiling crisis phenomenon part I: Nucleation and nucleate boiling heat transfer. *Exp. Therm. Fluid Sci.* **26,** 775–792 (2002).
3. Incropera, F. P. & DeWitt, D. P. *Fundamentals of Heat and Mass Transfer*. (John Wiley and Sons, Inc., 2002).
4. Rahman, M. M., Ölçeroğlu, E. & McCarthy, M. Role of Wickability on the Critical Heat Flux of Structured Superhydrophilic Surfaces. *Langmuir* **30,** 11225–11234 (2014).
5. Zuber, N. Hydrodynamic Aspects of Boiling Heat Transfer. (Research Laboratory, Los Angeles and Ramo-Woolridge Corporation, University of California, Los Angeles, CA, 1959).
6. De Gennes, P. G. Wetting: Statics and dynamics. *Rev. Mod. Phys.* **57,** 827–863 (1985).
